# Neural correlates of approach–avoidance tendencies toward physical activity and sedentary stimuli: An MRI study

**DOI:** 10.1162/IMAG.a.28

**Published:** 2025-06-06

**Authors:** Boris Cheval, Leonardo Ceravolo, Ophelia Zimmermann, Kinga Igloi, David Sander, Peter van Ruitenbeek, Matthieu P. Boisgontier

**Affiliations:** Univ Rennes, École normale supérieure de Rennes, VIPS ^2^ , Rennes, France; Swiss Center for Affective Sciences, University of Geneva, Geneva, Switzerland; Neuroscience of Emotion and Affective Dynamics Lab, Department of Psychology, University of Geneva, Geneva, Switzerland; Département de Réadaptation et Gériatrie, Service de Médecine Interne et Rédaptation University Hospital of Geneva, Geneva, Switzerland; Department of Fundamental Neurosciences, University of Geneva, Geneva, Switzerland; Department of Neuropsychology and Psychopharmacology, Faculty of Psychology and Neuroscience, Maastricht University, Maastricht, The Netherlands; School of Rehabilitation Sciences, Faculty of Health Sciences, University of Ottawa, Ottawa, Canada; Bruyère Health Research Institute, Ottawa, Canada; Institut du Savoir Monfort, Hôpital Monfort, Ottawa, Ontario, Canada

**Keywords:** automatic responses, executive function, physical activity, fMRI, reward system, action inhibition

## Abstract

Automatic tendencies toward physical activity and sedentary stimuli are involved in the regulation of physical activity behavior. However, the brain regions underlying these automatic tendencies remain largely unknown. Here, we used an approach–avoidance task and magnetic resonance imaging (MRI) in 42 healthy young adults to investigate whether cortical and subcortical brain regions underpinning reward processing and executive function are associated with these tendencies. At the behavioral level, results showed more errors when avoiding sedentary stimuli (i.e., avatars in a sitting position) than physical activity stimuli (i.e., avatars in a running position). At the brain level, avoiding sedentary stimuli was associated with more activation of the motor control network (dorsolateral-prefrontal cortex, primary and secondary motor cortices, somatosensory cortex). In addition, increased activation of the bilateral parahippocampal gyrus and local hypertrophy of the right hippocampus were associated with a stronger tendency to approach sedentary stimuli. Together, these results suggest that avoiding sedentary stimuli requires higher levels of behavioral control than avoiding physical activity stimuli.

## 1 Introduction

Behavioral automatic responses are characterized as unintentional, uncontrollable, efficient, and unaware (Bargh, 2014). Recent theoretical work suggests that these responses are essential in explaining the gap between intentions to be physically active and actual engagement in physical activity (Brand & Ekkekakis, 2018;Cheval & Boisgontier, 2021;Cheval, Radel, et al., 2018;Cheval et al., 2024;Conroy & Berry, 2017;Maltagliati et al., 2025). In particular, within the theoretical framework that explains human behavior as driven by two interacting types of processes, automatic and controlled (Strack & Deutsch, 2004), the theory of effort minimization in physical activity (TEMPA) argues that people have an automatic attraction to effort minimization (Cheval & Boisgontier, 2021). This attraction may lead individuals to be automatically drawn to sedentary opportunities that arise in their environment (Cheval & Boisgontier, 2024). TEMPA posits that (1) sedentary behaviors are rewarding and (2) avoiding sedentary behavior requires more executive control than approaching sedentary behavior or avoiding physical activity (Cheval & Boisgontier, 2021;Cheval, Radel, et al., 2018).

According to TEMPA’s first postulate, sedentary behavior should be intrinsically rewarding and provide motivational drive to favor that behavior. This drive may be characterized by activation of specific brain regions. However, current neural evidence for the rewarding or motivational value of sedentary behavior is unclear. Some studies support this first postulate (Jackson et al., 2014;Prévost et al., 2010). For example, obese women showed a reduced activation of reward brain areas than lean women when viewing images of physical activity, suggesting that higher effort is associated with lower reward (Jackson et al., 2014). In addition, the prospect of energetic expenses was associated with activation in the anterior cingulate cortex and anterior insula, which was interpreted as signaling higher perceived costs (Prévost et al., 2010). However, other studies challenge this first postulate. For example,Crémers et al. (2012)showed that brain areas associated with reward (e.g., insula, pallidum, caudate) and motor control (e.g., dorsolateral prefrontal cortex [DLPFC]) were activated during the mental imagery of brisk walking (compared with lying and standing conditions). Using a go/no-go task toward stimuli depicting physical activity and inactivity, no evidence of activation was shown in brain areas associated with reward processing (Kullmann et al., 2014). Finally, in studies using electroencephalography (EEG), reward-related brain activity showed no evidence supporting that sedentary behavior was rewarding (Cheval, Boisgontier, et al., 2019;Parma et al., 2023). In summary, the neural evidence regarding the rewarding or motivating value of sedentary behavior is inconsistent.

Building on TEMPA’s second postulate, it can be suggested that active avoidance (i.e., moving away from sedentary behavior) requires executive control, involving activation of associated brain areas. In contrast, passive avoidance (i.e., refraining from moving toward sedentary behavior) may specifically depend on inhibitory control. Studies consistently support this second postulate, indirectly validated by large-scale epidemiological studies showing the importance of cognitive function in facilitating and sustaining engagement in physical activity (Cheval, Boisgontier, et al., 2022;Cheval, Orsholits, et al., 2020;Cheval, Rebar, et al., 2019;Cheval et al., 2023;Csajbók et al., 2022;Daly et al., 2015;Sabia et al., 2017). EEG studies provide a more direct support for this postulate (Cheval et al., 2021;Cheval, Daou, et al., 2020;Cheval, Tipura, et al., 2018). For example, avoiding sedentary stimuli, compared with avoiding physical activity stimuli, was associated with larger evoked-related potentials in the frontal cortical areas (Cheval, Tipura, et al., 2018). Similarly, a study using a go/no-go task showed that passively avoiding stimuli representing sedentary behavior, compared with physical activity, was associated with larger evoked-related potentials in the frontocentral cortex (Cheval et al., 2021;Cheval, Daou, et al., 2020). However, the limited spatial resolution of EEG prevents these studies from precisely identifying the neural networks underlying these automatic responses.

To the best of our knowledge, only one functional magnetic resonance imaging (fMRI) study has been conducted to investigate brain areas potentially underlying executive control in the processing of physical activity and sedentary stimuli (Kullmann et al., 2014). The results of this study suggest that passively avoiding stimuli related to physical activity is associated with an increased demand on the inhibitory control system (e.g., prefrontal cortex) in patients with anorexia nervosa (Kullmann et al., 2014). However, this association may be explained by the fact that patients with anorexia nervosa often report excessive levels of physical activity (Davis et al., 1997), limiting the generalizability of the results to the general population, where a reverse pattern may be expected (Cheval et al., 2021;Cheval, Daou, et al., 2020). Therefore, using fMRI to investigate the brain regions underlying executive control in the processing of physical activity and sedentary stimuli in healthy adults is warranted.

### 1.1 The present study

The aim of the present study was to investigate whether brain regions involved in reward processing and executive control are activated when an individual is exposed to stimuli depicting different levels of physical activity, as measured by fMRI. Specifically, based on the postulates of TEMPA and previous work, this study focused on brain regions associated with reward processing, such as orbitofrontal cortex, amygdala, and ventral striatum (Corbit & Balleine, 2011;Gottfried et al., 2003;Knutson et al., 2001;Prévost et al., 2012;Roesch & Olson, 2004;Schultz et al., 2000), and with executive control, such as DLPFC, inferior frontal cortex, presupplementary motor area, and basal ganglia (striatum and subthalamic nucleus) (Aron et al., 2007,^2014^;Zandbelt & Vink, 2010). To this end, healthy young participants performed an “implicit” approach–avoidance task using stimuli depicting sitting, standing, and running avatars during fMRI. In addition, we analyzed how variations in the shape of subcortical structures are associated with a tendency to avoid physical activity or to approach sedentary behavior.

### 1.2 Hypotheses

At the behavioral level, we hypothesized shorter reaction times and/or fewer errors when approaching sedentary behavior, as illustrated by sitting avatars, than when approaching physical activity, as illustrated by running avatars (HB1). In contrast, we hypothesized longer reaction times and/or more errors when avoiding sedentary stimuli than when avoiding physical activity stimuli (HB2).

At the brain level, we hypothesized increased activity in brain areas associated with reward when approaching compared with avoiding sedentary stimuli (HN1) (contrast: approach sitting > avoid sitting). In addition, we hypothesized increased activity in brain areas involved in executive control when avoiding compared with approaching sedentary stimuli (HN2) (contrast: avoid sitting > approach sitting) and when avoiding sedentary stimuli compared with avoiding physical activity stimuli (HN3) (contrast: avoid sitting > avoid running). Moreover, we hypothesized that brain activity differences observed in HN3 would not be observed with stimuli depicting light physical activity (i.e., standing avatars) (contrast: avoid sitting> avoid standing) (HN4). Previous studies have observed associations between specific areas of subcortical brain structures and a variety of functions, including intelligence (Burgaleta et al., 2014), circadian rhythm function (Xu et al., 2023), and sensorimotor control (Boisgontier, Cheval, et al., 2016;Boisgontier, van Ruitenbeek, et al., 2016). Based on these observations, we hypothesized that local spatial variability (i.e., local dip or bulge) in subcortical brain structures involved reward processing (i.e., nucleus accumbens, pallidum) and habits (i.e., caudate, putamen) would be associated with the tendency to avoid physical activity and approach sedentary behavior. Other subcortical areas were part of an exploratory analysis.

## 2 Materials and Methods

### 2.1 Participants

To estimate the sample size required for adequate power (90%) with an alpha level set at 5%, we conducted an a priori power analysis using G*Power 3.1 (Faul et al., 2009). We performed a power analysis for a repeated-measures ANOVA with a small-to-medium effect size (Cohen’s*d*= 0.40). We set groups to 1, measures to 6 (action, stimuli), correlations between repeated measures to 0.5, and non-sphericity to 1. The power calculation estimated a required*N*of 36, but we aimed to recruit 45 to account for potential data loss due to collection issues.

Exclusion criteria included a history of psychiatric, neurological, or severe mental disorders; use of psychotropic medications, alcohol, or illicit drugs at the time of the study; and any MRI contraindications. In addition, participants were screened to include only those who were right handed (Oldfield, 1971), could understand French, were >18 years of age, and were free of any medical conditions that would prohibit physical activity without supervision. Smokers were abstinent from tobacco for at least 1.5 h prior to scanning to reduce the effects of nicotine on the blood oxygen-dependent level (BOLD) signal (Jacobsen et al., 2002). Participants read and completed a written informed consent form. The study was approved by the ethics committee of the Canton of Geneva, Switzerland (CCER-2019-00065). All participants gave informed consent before participating in the study and were compensated with 100 Swiss francs for their participation.

Participants were recruited in two ways. First, we used a database maintained by one of the coauthors (KI) that included individuals who had previously expressed interest in participating in future studies and had agreed to be recontacted. Second, we posted flyers at the University of Geneva, inviting individuals to participate in an fMRI research study. In total, 47 healthy volunteers were recruited. Data from five participants were excluded due to the inability to enter the MRI scanner (e.g., presence of piercings, tattoos, or copper intrauterine device). The final sample consisted of 42 participants (31 women, 23.0 ± 3.5 years; body mass index = 21.4 ± 3.0 kg.m^-2^).

### 2.2 Experimental paradigm

At least 2 days prior to the experimental session, participants completed an online questionnaire measuring laterality (Edinburgh Handedness Inventory) (Oldfield, 1971), usual level of physical activity and sedentary behavior (International Physical Activity Questionnaire) (Craig et al., 2003), motivation for physical activity (i.e., attitudes, intentions, and motivation), exercise dependence (Griffiths et al., 2005), approach–avoidance temperament (Elliot & Thrash, 2010), and demographics (age, sex, height, and weight). Prior to entering the MRI scanner, participants completed a checklist to ensure that they met the requirements to perform a task in the MRI scanner and a questionnaire to assess potential confounding variables (e.g., caffeine, alcohol, and cigarette consumption). An MRI assistant then equipped the participants with the physiological measurements (i.e., respiratory rate, galvanic response, cardiac rhythm) and positioned them in the scanner. Participants were instructed on how to behave during the experiment (e. g., move as little as possible, especially the head). Both foam padding and a strap across the participant’s forehead were used to minimize head movement.

To assess approach–avoidance tendencies and the associated neural activations, participants completed the Visual-Approach/Avoidance-by-the-Self-Task (VAAST) (Rougier et al., 2018) during fMRI. The task was presented using E-Prime (beta 5.0 version) software (Psychology Software Tools Inc.). The MRI sequences included the first two functional runs of the VAAST (8 min each), a T1-weighted scan (5 min), the last two functional runs (8 min each), a T2-weighted scan (5 min), a resting state (8 min) and a reward localizer task (13 min). Finally, participants were paid and debriefed. The entire session lasted approximately 100 min.

#### 2.2.1 Stimuli

Using Unity software, we created images of avatars in sitting, standing, and running positions to represent increasing intensities of physical activity: sedentary activity, light physical activity, and mild-to-moderate physical activity, respectively. The images were designed to maintain identical color, brightness levels, and comparable visual complexity. Specifically, a set of 195 images containing 14 sitting, standing, and running avatars (50% woman) was tested in a pilot study in which 105 participants were asked to rate a random set of 65 images. The participants were asked to rate the extent to which they associated each stimulus with “movement and physically active behavior” (vs. “rest and physically inactive behavior”) using two Visual Analogue Scales (VAS1: “Please indicate the extent to which you think this image is associated with a behavior that requires: 0 = No physical exertion at all, 100 = A lot of physical exertion”; VAS2: “Please indicate how closely this image is associated with: 0 = Resting, sedentary behavior, 100 = Moving, very active behavior”). In addition, the participants rated the credibility (“How realistic do you think this person’s behavior is? Realistic means that the images may resemble a real-life behavior”; on a VAS from 0 = behavior not at all realistic; 100 = Behavior very realistic) and the likeability of each image (“How likeable/sympathetic do you find the person in this image? For example, would you like to talk to him/her”; on a VAS from 0 = Very unpleasant/antipathetic, 100 = Very pleasant/sympathetic).

The purpose of the pilot study was twofold. First, to ensure that the selected images reflected the concepts of interest (i.e., movement and physical activity vs. rest and physical inactivity). Second, to test whether the selected images were equivalent in terms of credibility and pleasantness across categories (i.e., movement vs. rest). Based on the results of the pilot study, we selected a total of 84 images that included 12 avatars (50% woman) in 7 activities (3 sitting, 1 standing, and 3 running). Each avatar was represented in the seven positions to ensure a strict equivalence between the conditions.

In this pilot study, the running stimuli were associated with a significantly higher level of physical effort (72.4 ± 2.52) than the sitting (17.45 ± 2.98,*p*< 0.001) and standing stimuli (38.15 ± 2.01,*p*< 0.001). Similarly, the sitting stimuli were associated with a significant lower level of physical effort compared with the standing stimuli (*p*< 0.001). On average, the images were rated as credible (81.48 ± 3.10) and had a moderate level of pleasantness (55.72 ± 7.92). Results showed no evidence of a difference in credibility between running (81.63 ± 2.83) and sitting stimuli (80.24 ± 2.86) (*p*= 0.089), but standing stimuli (84.70 ± 2.12) were rated as more credible than running (*p*= 0.004) and sitting stimuli (*p*< 0.001). No significant differences in pleasantness were observed between the stimuli (55.15 ± 8.16, 56.20 ± 8.97, and 56.14 ± 7.53 for sitting, standing, and running stimuli, respectively;*p*= 0.850). These results demonstrated the validity of the stimuli in terms of their association with the level of physical effort and confirmed that these stimuli were mostly equivalent in terms of pleasantness and credibility. A sample of the stimuli used in each category is provided inSupplementary Material 1. Additionally, the full set of stimuli is publicly available on Zenodo (Cheval, Ceravolo, Igloi, Zimmermann, et al., 2025).

#### 2.2.2 The Visual-Approach/Avoidance-by-the-Self-Task (VAAST)

An adapted version of the VAAST was used to measure automatic approach–avoidance tendencies toward physical activity and sedentary behaviors (Rougier et al., 2018). Compared with other approach–avoidance tasks such as the manikin task (Cheval et al., 2014,^2015^;Krieglmeyer & Deutsch, 2010), the VAAST has been shown to produce larger and more replicable effects. During the task, participants were asked to respond to the format (i.e., portrait vs. landscape format) of the images depicting avatars in active (i.e., running), inactive (i.e., sitting), and neither active nor inactive (i.e., standing) positions by pressing the “move forward” or “move backward” button three times on an MR-compatible response box (Current Designs Inc., Philadelphia, PA, USA), which was placed beneath the participant’s fingers. Participants were instructed to approach the image when it appeared in a portrait format, and to avoid it when it appeared in a landscape format (the rule was counterbalanced across participants). The visual environment was dynamically adjusted based on participants’ responses: zooming in to simulate an approach movement and zooming out to simulate an avoidance movement. Specifically, to enhance the impression of forward or backward movement, the size of the visual scene increased or decreased by 30% immediately after each button press, creating the impression of moving toward or away from the stimulus. The video of this task is available on Zenodo (Cheval, Ceravolo, Igloi, Zimmermann, et al., 2025).

**Fig. 1.**
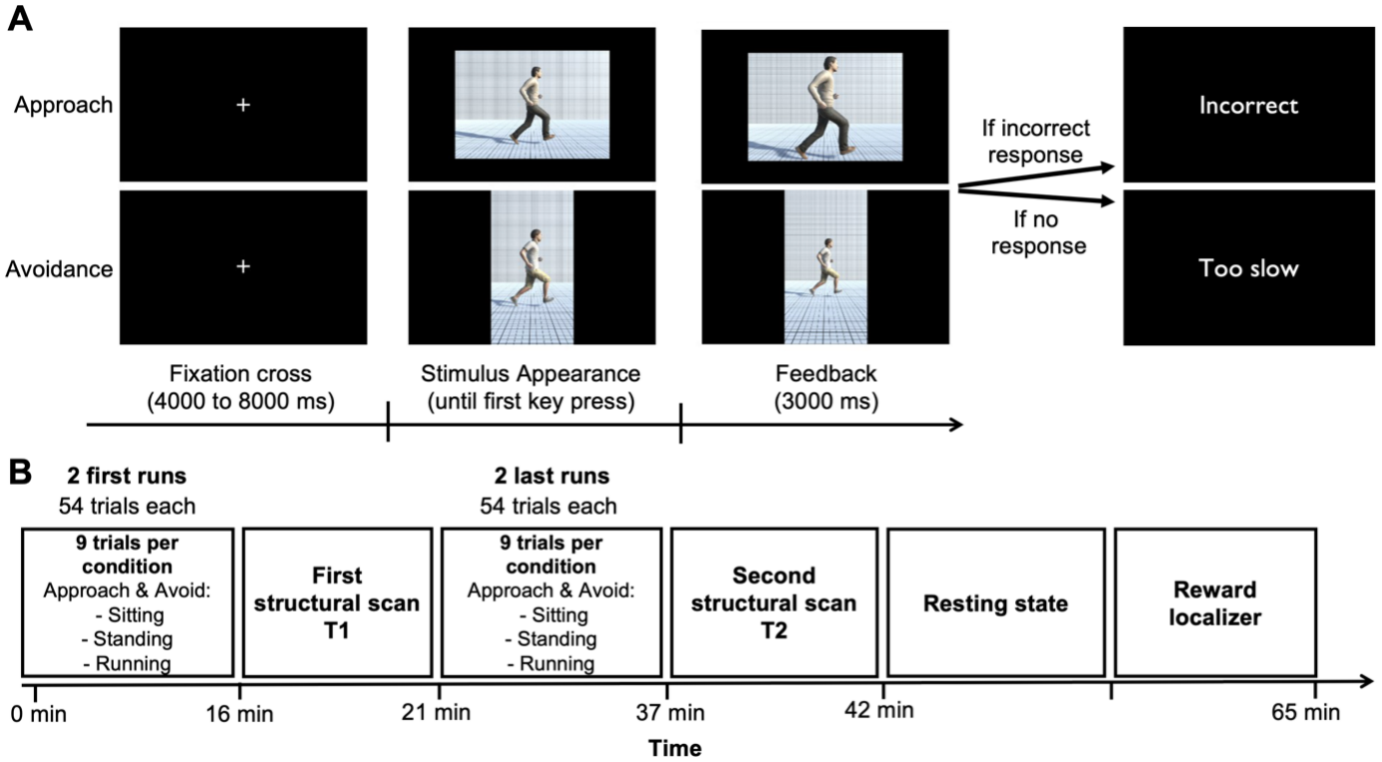
Experimental paradigm. (A) The approach–avoidance task. Participants were instructed to quickly approach or avoid images depending on their format (i.e., portrait vs. landscape format). The six conditions (i.e., approach sitting, approach standing, approach running, avoid sitting, avoid standing, avoid running) were pseudorandomized across the runs. (B) Procedure. Participants were asked to complete four runs of the approach–avoidance task. Each run was composed of 54 trials, including 9 trials within each of the 6 conditions.

The VAAST was administered in four runs. Each run consisted of 54 trials, for a total of 216 trials. Each run included an equal number of trials (i.e., nine) for each of the six conditions representing the interaction between the two main factors: Type of action and Type of stimuli (i.e., approach sitting, approach standing, approach running, avoid sitting, avoid standing, avoid running). The total number of trials per condition was 36, which is twice as many as the number of trials that have shown fair to good test–retest reliability of neural responses in an approach–avoidance conflict task (McDermott et al., 2021). To minimize fatigue and order effects during the scanning session, we implemented the following counterbalancing procedures: randomizing trial order within each run, pseudorandomizing stimuli across the runs, and providing short breaks between runs to maintain participant engagement. To avoid expectancy effects, we varied the duration of the fixation cross (interstimulus interval; 4–8 s) in each trial (Fig. 1).

### 2.3 Behavioral analysis

Statistical analyses of the behavioral data (i.e., reaction times and errors) were conducted using R, version 4.4.2 (R Core Team, 2019). Specifically, mixed-effects models (Baayen et al., 2008;Boisgontier & Cheval, 2016) were used via the lme4 and lmerTest packages (Bates et al., 2014;Kuznetsova et al., 2015) to account for the cross-random structure of the current data (i.e., a random sample of participants crossed with a random sample of stimuli) and thereby correctly estimate the parameters.

To examine reaction times, the linear mixed-effects models included as fixed factors the type of stimuli (i.e., sitting, standing, running) and the type of action (i.e., approach, avoidance), and an interaction between these two fixed factors. Participants and stimuli (i.e., images) were specified as random factors, and the model included random effects of the type of action, the type of stimuli. These random parameters allowed the effects of the fixed factors on the reaction times to vary across participants. The equation of the model was as follows:

$$\begin{array}{l} {\text{Reaction Time}}_{\text{ijk}}\;={\text{ß}}_{0}\;+{\text{ß}}_{1}{\text{ Action}}_{\text{i}}+{\text{ß}}_{2}{\text{ Stimuli}}_{\text{j}}\;\\ \;\;\;\;\;\;\;\;\;\;\;\;\;\;\;\;\;\;\;\;\;\;\;\;\;\;\;\;\;\;\;\;\;\;\;\;\;\;\;\;\;\;\;\;\;\;\;+{\text{ß}}_{3}{\text{ (Action}}_{\text{i}}\;\times {\text{Stimuli}}_{\text{j}}\text{)}+\;{\text{ß}}_{4}{\text{ Age}}_{\text{k}}\\ \;\;\;\;\;\;\;\;\;\;\;\;\;\;\;\;\;\;\;\;\;\;\;\;\;\;\;\;\;\;\;\;\;\;\;\;\;\;\;\;\;\;\;\;\;\;\;+{\text{ß}}_{5}{\text{ Sex}}_{\text{k}})\;\;+\;{\text{ß}}_{6}{\text{ Body Mass Index}}_{\text{k}}\\ \;\;\;\;\;\;\;\;\;\;\;\;\;\;\;\;\;\;\;\;\;\;\;\;\;\;\;\;\;\;\;\;\;\;\;\;\;\;\;\;\;\;\;\;\;\;\;+\;u_{0\text{k}}\;+\;u_{1\text{k}}\text{ Action}+u_{2\text{k}}\text{ Stimuli}+v_{\text{oj}}\;\times \;\varepsilon_{\text{ijk}.} \end{array}$$

In this equation,*Reaction Time_ijk_*represents the outcome (reaction time) for Subject*k*, Action*i*, and Stimulus*j*,*β0*is the fixed intercept,*β1, β2,*and*β3*are the fixed effects for Action, Stimuli, and their interaction.*β4*,*β5*, and*β6*are the fixed effects for age, sex, and body mass index (z-scored).*u_0k_*is the random intercepts for Subject*k*,*u_1k_*and*u_2k_*represent the random slopes for Action and Stimuli, respectively, for Subject*k*. v_oj_is the random intercept for images.*ε_ijk_*is the residual error term.

For exploratory analyses, we conducted additional models including three-way interactions of usual physical activity level, sedentary craving state, and physical activity craving state with stimulus type and action type (Supplementary Material 2). The latter models allowed us to examine the extent to which dispositional or situational factors may alter participants’ reaction times to approach (vs. avoid) sitting, standing, and running stimuli, as expected by TEMPA (Maltagliati et al., 2024). The same models were applied to errors, except that linear mixed-effects models were replaced by logistic mixed-effects models to predict the probability of making an error.

To reduce convergence problems, each model was optimized using the default BOBYQA optimizer (Powell, 2009), the Nelder–Mead optimizer (Nelder & Mead, 1965), the nlimb optimizer from the optimx package (Nash & Varadhan, 2011), and the L-BFGS-B optimizer (seeCheval, Bacelar, et al., 2020;Cheval et al., 2021;Cheval, Maltagliati, et al., 2022;Frossard & Renaud, 2019, for similar procedure).*P*values for the global effect of the factors and their interaction were reported using likelihood ratio tests comparing models with and without the fixed factors included in the models. Statistical assumptions associated with mixed-effects models (i.e., normality of the residuals, linearity, multicollinearity, and undue influence) were met.

### 2.4 MRI data acquisition

High-resolution imaging data were acquired on a 3-Tesla whole-body MRI system (Magnetom Tim Trio, Siemens, Erlangen, Germany) equipped with a 12-channel head coil. We used multislice echo planar imaging sequences. For each participant and for each run of the experimental task, 79 functional 2D T2*-weighted echo planar image volumes (EPIs; voxel size = 2.5 × 2.5 × 2.5 mm, 48 slices, TR = 600 ms, TE = 32 ms, matrix = 84 × 84, FoV = 210 × 210 mm, in-plane resolution = 64 × 64, FA = 52 degrees) were acquired. Thus, an average of 900 volumes of 48 slices were acquired for each participant. The 192 high-resolution 3D T1-weighted structural images (1 mm^3^isotropic voxels, TR = 1900 ms, TE = 2.27 ms, FA = 9 degrees, FoV = 256 × 256 mm) were acquired using a magnetization-prepared rapid acquisition gradient echo sequence.

### 2.5 fMRI data preprocessing

Functional images were analyzed using Statistical Parametric Mapping software (SPM12, Wellcome Trust Centre for Neuroimaging, London, UK). Preprocessing steps included realignment to the first volume of the time series, normalization to the Montreal Neurological Institute (MNI) space (Collins et al., 1994), and spatial smoothing with an isotropic Gaussian filter of 8 mm full width at half maximum. To remove low-frequency components, we used a high-pass filter with a cutoff frequency of 1/128 Hz.

### 2.6 fMRI data analysis

Data were analyzed using general linear modeling (GLM) as implemented in SPM12 (https://www.fil.ion.ucl.ac.uk/spm/). For the first-level analyses of the experimental task, correctly scored trials of our conditions of interest (design matrix conditions: 1. approach running; 2. avoid running; 3. approach sitting; 4. avoid sitting; 5. approach standing; 6. avoid standing) and trial-level reaction times were modeled by fitting a boxcar function at the onset of the feedback screen convolved with the canonical hemodynamic response function for 3 s (duration of the feedback screen). An additional column was added to the design matrix, containing error trials (wrong response trials) and trials for which response times were outside the bounds of percentiles 2 and 98 to remove trials where participants either pressed the button too quickly to see the image or did not respond at all. These types of trials were concatenated into a single column per run and only contained on average two trials per run. The design matrix included the 6 columns of interest with the corresponding 6 columns of reaction times and the “error” trials and the 6 realignment parameters to account for movement in the data, for a total of 19 columns per run per participant. The six motion correction parameters were included as regressors of no interest to minimize false-positive activations due to task-correlated motion, and framewise displacement did not differ significantly between conditions (*p*> 0.05). The four runs were modeled in a single first-level design matrix with runs separated as four different sessions of one participant. Contrasts were computed with the main effect of each of the six conditions of interest (value of “1”) inversely correlating with reaction times for each condition (value of “-1”).

Whole brain group-level statistics were then performed using a 252-lines flexible factorial analysis, in which the first-level simple effects were implemented (42 participants * 6 conditions = 252 files/lines). The model, therefore, included the factors Participants, Type of action (i.e., approach, avoidance), and Type of stimuli (i.e., sitting, standing, running). Their interaction was also tested. Independence was set to “true” for the Participants factor and to “false” for the remaining within factors. Variance estimation was set to “unequal” for all factors because homoscedasticity criteria cannot usually be met for fMRI data (default setting in SPM12). Group-level results of our final contrasts of interest were then corrected for multiple comparisons using a voxel-wise threshold of*p*< 0.05 with false discovery rate (FDR) correction, with q = 0.05, defined as the proportion of false positives (Type I errors) among all rejected tests (Bennett et al., 2009), as this approach has shown better ratios of true to false positives than the family-wise error rate (FWER) when the signal-to-noise ratio is low (Lindquist & Mejia, 2015), which may be the case here given the 600-ms TR. Given our voxel size of 2.5 × 2.5 × 2.5 mm (15.625 mm³ per voxel) and the approximate volume of our smallest structure of interest, the hippocampus (3,250) mm³ (Pruessner et al., 2000), we applied a cluster extent threshold of 10 voxels, representing approximately 5% of the hippocampal volume, to balance the risk of false positives while maintaining sensitivity. For all analyses, regions were labeled using the latest version of the Automated Anatomical Labelling Atlas (“AAL3”) (Rolls et al., 2020) and rendered on semi-inflated brains from the CONN toolbox (http://www.nitrc.org/projects/conn).

### 2.7 Vertex analysis

An exploratory analysis was conducted to examine a potential association between the shape of subcortical structures (i.e., nucleus accumbens, amygdala, caudate, hippocampus, pallidum, putamen, and thalamus) and approach–avoidance tendencies toward sedentary behavior or physical activity. For reaction times and errors, the tendency to approach rather than avoid sedentary behavior is represented by the difference between avoiding and approaching sitting stimuli (i.e., avoid sitting–approach sitting). The tendency to avoid physical activity rather than sedentary behavior is represented by the difference between avoiding sitting stimuli and avoiding running stimuli (i.e., avoid sitting–avoid running).

The individual structure’s shape is represented by a mesh consisting of vertices. The vertices represent points on the mesh. These vertices are compared with an average mesh. The quantitative differences between the vertices and the mesh represent inward (i.e., local dip or atrophy) and outward (i.e., local bulge or hypertrophy) deformations of the individual structure. To obtain these measures, T1-weighted images were reoriented to standard orientation. Next, structures were segmented from the T1-weighted images using FMRIB’s Integrated Registration Segmentation Toolkit (FSL FIRST;Patenaude et al., 2011) in FSL version 6.0.7.13 (Jenkinson et al., 2012;Smith et al., 2004;Woolrich et al., 2009). As a sub-step, T1 images were registered to normalized space. Accuracy of the registrations were visually inspected for all participants using the “slicesdir” command to create coronal, sagittal, and horizontal slices. Subsequently, vertex analysis (FSL) was used to indicate the exact location of the relationship between subregional gray matter structure and behavioral tendencies. The vertices represent the signed, perpendicular distance from the average surface. Negative and positive values reflect inward (i.e., local atrophy) and outward (i.e., local expansion) deformation of the structures, respectively. FSL FIRST vertex analysis (Patenaude et al., 2011) restricts topology of the structures and preserves inter-participant vertex correspondence, enabling a vertex-wise comparison of differences between conditions in the association with behavioral tendencies. The regression models using behavior tendencies predicting structural deviations from the mesh representing average shape were created and tested for significance using permutation-based non-parametric tests (FSL randomize, 10,000 draws,*p*< 0.05, TFCE applied, FWE corrected) (Smith & Nichols, 2009).

### 2.8 Functional and effective connectivity analyses

Undirected connectivity analyses were conducted to explore potential relationships between regions of interest. Specifically, we used task-related generalized psychophysiological interactions (gPPIs) as implemented in the CONN toolbox (version 22.a) in Matlab 9.0 (MathWorks, Inc., Natick, MA, USA) (Whitfield-Gabrieli & Nieto-Castanon, 2012). The gPPIs illustrate the level of task-modulated connectivity between seed regions/voxels, by computing a separate multiple regression model for each target seed/voxel. Regression coefficient maps were compared across conditions to examine the interaction between psychological and physiological factors. This comparison was conducted using bivariate correlations for functional connectivity maps and bivariate regression analyses for effective connectivity maps, based on the predefined contrasts of interest. Maps of regression coefficients were compared for each condition and according to the above-mentioned contrasts of interest.

## 3 Results

### 3.1 Descriptive results

Table 1shows the characteristics of the participants and reports the reaction times to approach and avoid stimuli depicting sitting, standing, and running avatars, as well as the approach bias scores (i.e., reaction times to avoid–reaction times to approach) for each type of stimulus. On average, reaction times within each condition were <700 ms and showed strong correlations across conditions (Pearson’s*R*’s between 0.83 and 0.95,*p*’s < 0.001). These correlations indicate that for a given participant, reaction times in one condition were strongly associated with reaction times in the other conditions. Error rates were on average about 5% (±6%) for avoiding running stimuli, 6% (±9%) for approaching standing stimuli, and about 7% for the other conditions (standard deviations ranged from 6% to 9%).

**Table 1.** Descriptive statistics.

N = 42	Mean	SD
Age (years)	23.0	3.5
Gender (number; %)		
Women	31	74%
Men	11	26%
Body mass index	21.4	3.0
Craving for sedentary behaviors	4.3	1.6
Craving for physical activity behaviors	3.7	1.7
Usual level of physical activity (min per week)	285.9	293.1
**Reaction times (ms)**		
Approach running	666.5	111.4
Approach standing	668.7	117.5
Approach sitting	677.4	116.5
Avoid running	659.6	102.8
Avoid standing	659.4	107.6
Avoid sitting	669.7	114.3
**Approach biases (ms)**		
Approach bias toward running	-6.9	47.2
Approach bias toward standing	-9.3	65.6
Approach bias toward sitting	-7.7	65.7
**Errors**		
Approach running	7%	8%
Approach standing	6%	9%
Approach sitting	7%	7%
Avoid running	5%	6%
Avoid standing	7%	8%
Avoid sitting	7%	8%

*Notes.*SD = standard deviation; ms = milliseconds; min = minutes.

### 3.2 Reaction times and error rates in the approach–avoidance task

#### 3.2.1 Reaction times

The results of the linear mixed-effects models showed no main effect of stimulus type (*p-*value for global effect = 0.164) or action type (*p*-value for global effect = 0.160). The two-way interaction between stimulus type and action type was not significant (*p*-value for global effect = 0.965). Simple effects tests further confirmed that reaction times to approach (vs. avoid) running stimuli were not statistically different from reaction times to approach (vs. avoid) sitting stimuli (*p*= 0.851) (Table 2). Similarly, reaction times to approach (vs. avoid) standing stimuli were not statistically different from reaction times to approach (vs. avoid) sitting stimuli (*p*= 0.802) or running stimuli (*p*= 0.661).

**Table 2.** Results of the linear mixed-effects models predicting the reaction times as a function of action type (approach vs. avoidance) and stimulus type (sitting vs. standing vs. running).

**N** **=** 40*	b (CI)	*p*
**Fixed Effects**		
Intercept	**666.1 (627.9; 704)**	**<0.001**
**Stimuli (ref. physical activity)**		
Standing	2.7 (-11.7; 17.2)	0.711
Sitting	7.9 (-6.0; 21.9)	0.267
**Action (ref. approach)**		
Avoidance	-7.0 (-23.6; 9.6)	0.410
**Stimuli (ref. physical activity)** **x** **Action (ref. approach)**		
Avoidance x Standing	-4.1 (-11.7; 14.2)	0.661
Avoidance x Sitting	-1.8 (-22.4; 16.6)	0.851
**Covariates**		
Age	-12.3 (-46.4; 21.7)	0.483
Sex	-5.1 (-78.3; 68.1)	0.892
BMI	1.3 (-33.0; 35.6)	0.940
**Random Effects**		
**Participants**		
Intercept	10710.07	
Sitting	54.48	
Standing	8.24	
Action avoid	1109.29	
Corr. (Intercept, sitting)	0.07	
Corr. (Intercept, standing)	0.97	
Corr. (Intercept, action avoidance)	-0.30	
Corr. (sitting; standing)	-0.16	
Corr. (sitting; action avoidance)	0.93	
Corr. (standing; action avoidance)	-0.51	
**Stimuli**		
Intercept	94.87	
Residual	28551.56	
R ^2^	Conditional = 0.005Marginal = 0.275	

*Notes*. 95CI = confidence intervals at 95%; BMI = body mass index. *Two participants were not included in the analyses because they were an issue regarding the recording of their behavioral data.

#### 3.2.2 Errors

**Fig. 2.**
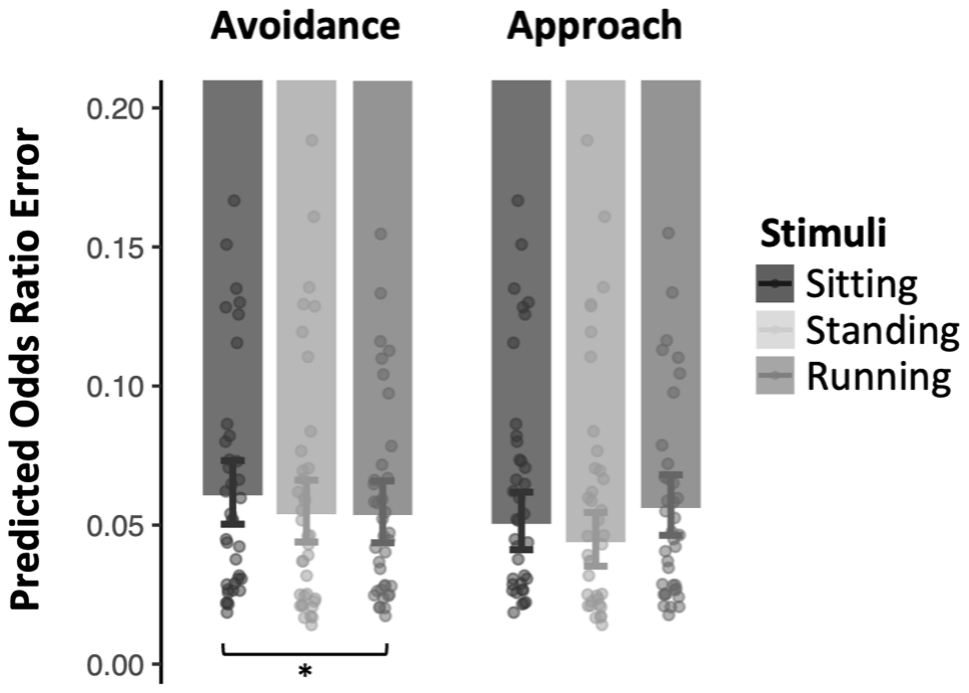
Results of the logistic mixed-effects models. Estimated odds ratios of a failure to avoid or approach sitting, standing, and running stimuli. Dots represent mean response times for each participant as a function of stimulus type. Error bars represent the standard errors around the mean. * Indicates statistically significant differences (*p*< .05).

The results of the logistic mixed-effects models showed no main effect of stimulus type (*p-*value for global effect = 0.784) or action type (*p*-value for global effect = 0.995). However, although the main effect of the interaction between stimulus type and action type was only marginal (*p*-value for global effect = 0.091), the results showed that the probability of error when avoiding (vs. approaching) running stimuli was statistically different from the probability of error when avoiding (vs. approaching) sitting stimuli (OR = 1.64, 95%CI = 1.06 – 2.54,*p*= 0.025). Participants made more errors when avoiding sitting stimuli than when avoiding running stimuli. No difference was observed in the approach condition (Fig. 2). The same pattern of effect was found between standing and running stimuli (OR = 1.57, 95%CI = 1.01 – 2.43,*p*= 0.044) (Table 3).

**Table 3.** Results of the logistic mixed-effects models predicting the risk of error in the approach–avoidance task as a function of action type (approach vs. avoidance) and stimuli type (sitting vs. standing vs. running).

**N** **=** **40**	OR (CI)	*p*
**Fixed Effects**		
Intercept	**0.06 (0.04; 0.08)**	**<0.001**
**Stimuli (ref. Running)**		
Standing	0.80 (0.59; 1.08)	0.149
Sitting	0.85 (0.63; 1.16)	0.308
**Action (ref. approach)**		
Avoidance	0.73 (0.53; 1.03)	0.071
**Stimuli (ref. physical activity)** **x** **Action (ref. approach)**		
Avoidance x standing	**1.57 (1.01; 2.43)**	**0.044**
Avoidance x sitting	**1.64 (1.06; 2.54)**	**0.025**
**Covariates**		
Age	1.04 (0.77; 1.40)	0.798
Sex	0.94 (0.49; 1.81)	0.852
BMI	0.90 (0.67; 1.23)	0.516
**Random Effects**		
**Participants**		
Intercept	0.68	
Sitting	0.01	
Standing	0.02	
Action avoid	0.01	
Corr. (Intercept, sitting)	0.65	
Corr. (Intercept, standing)	1.00	
Corr. (Intercept, action avoidance)	-0.37	
Corr. (sitting; standing)	0.57	
Corr. (sitting; action avoidance)	-0.95	
Corr. (standing; action avoidance)	-0.28	
**Stimuli**		
Intercept	null	
R ^2^	Conditional = 0.006Marginal = 0.192	

*Notes*. OR = odds ratio; 95CI = confidence intervals at 95%; BMI = body mass index. Note that the models estimated a null variance for the random intercept of the stimuli. The models with or without this parameter lead to consistent results. *Two participants were not included in the analyses because they were an issue regarding the recording of their behavioral data.

### 3.3 Physical activity engagement and craving for physical activity

#### 3.3.1 Reaction times

Results showed no evidence suggesting that usual physical activity engagement or craving for physical activity significantly moderated the effect of action, stimulus type, or the interaction between these two factors (Supplementary Material 3). However, results showed that reaction time differences between responses to sitting and running stimuli were moderated by the state of craving for sedentary behaviors (b = -22.0, 95%CI = -35.0 – -9.0,*p*< 0.001). Participants responded faster to sitting than to running stimuli when their craving for sedentary behavior was high and were slower when their craving for sedentary behavior was low.

#### 3.3.2 Errors

Results showed no evidence suggesting that usual physical activity, craving for physical activity, or craving for sedentary behavior moderated the effect of action, stimulus type, or the interaction between these two factors (Supplementary Material 4).

### 3.4 Neural activity associated with the avoidance of sedentary stimuli

#### 3.4.1 Approach sitting > avoid sitting (HN1)

**Fig. 3.**
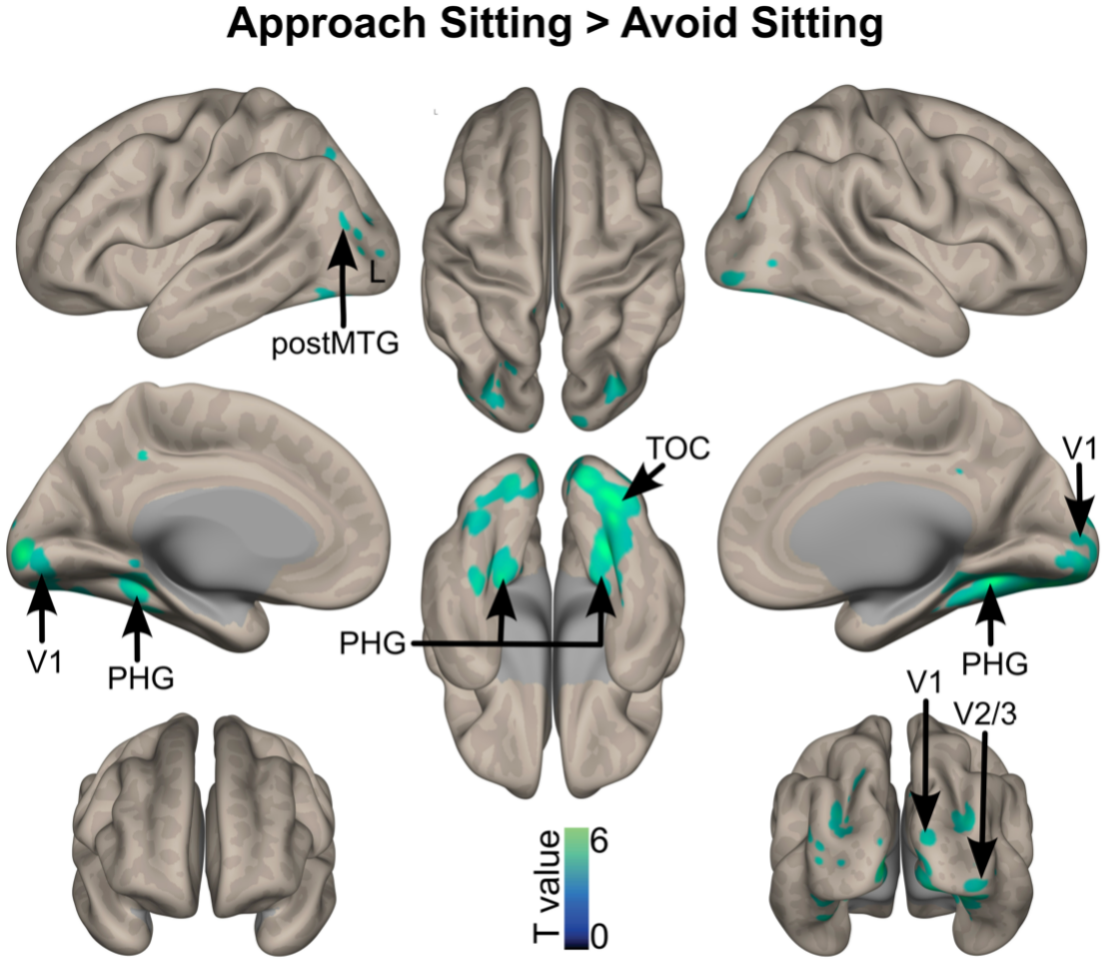
Brain activations when approaching versus avoiding sedentary stimuli, corrected for multiple comparisons (whole-brain voxel-wise FDR, q = 0.05, k > 10 voxels). The color bar represents the statistical T value. V1: primary visual cortex; V2/3: secondary visual cortex; postMTG: posterior part of the middle temporal gyrus; TOC: temporo-occipital cortex; PHG: parahippocampal gyrus. L: left hemisphere; R: right hemisphere.

More activation was observed in the left posterior middle temporal gyrus, bilateral parahippocampal gyrus, primary and secondary visual cortex when participants approached compared with avoid sitting stimuli (Fig. 3).

#### 3.4.2 Avoid sitting > approach sitting (HN2)

**Fig. 4.**
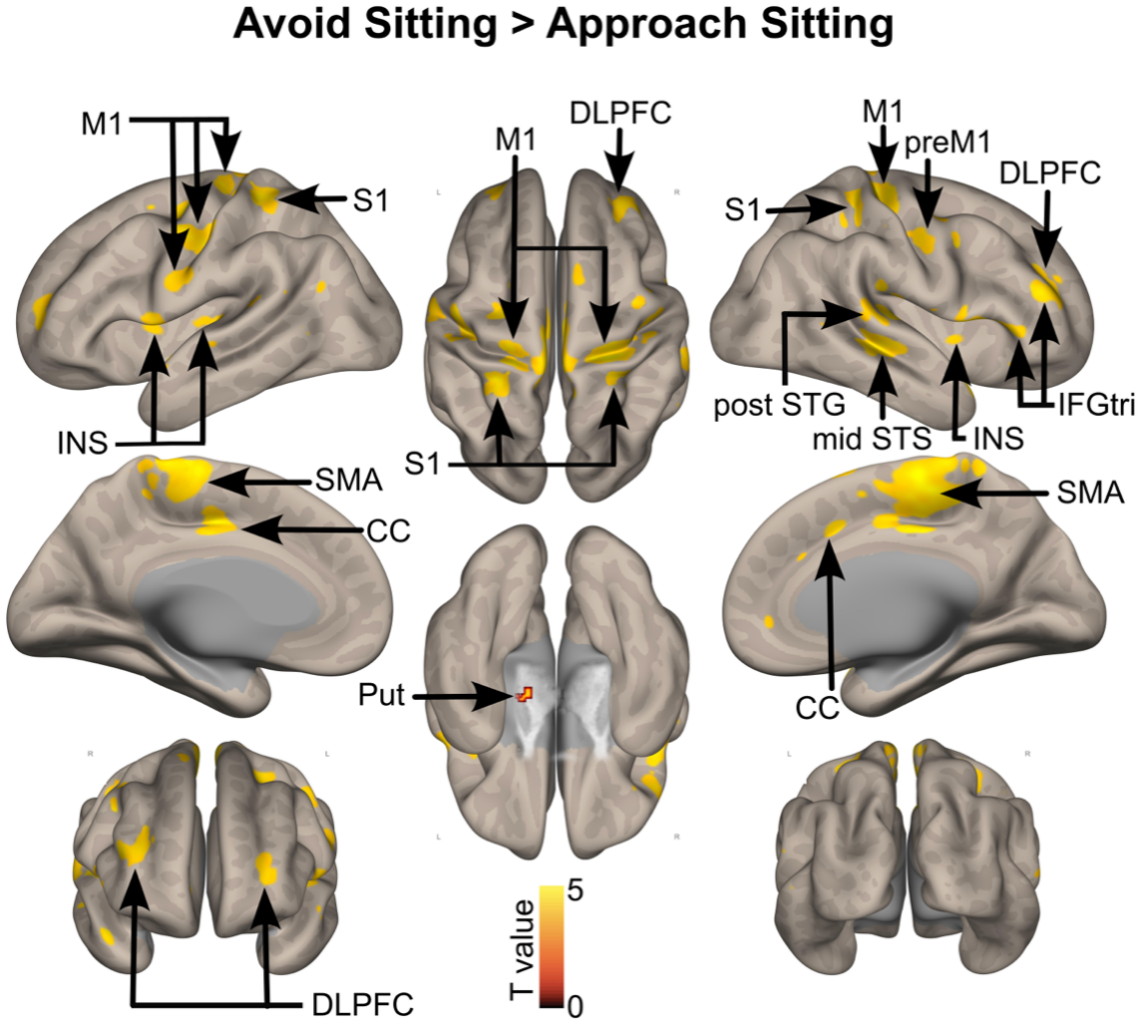
Brain activations when avoiding versus approaching sedentary stimuli, corrected for multiple comparisons (whole-brain voxel-wise FDR, q = 0.05, k > 10 voxels). The color bar represents the statistical T value. postSTG: posterior part of the superior temporal gyrus; midSTS: mid part of the superior temporal sulcus; M1: primary motor cortex; S1: primary somatosensory cortex; INS: insula; DLPFC: dorsolateral prefrontal cortex; IFGtri: inferior frontal gyrus*pars triangularis*; SMA: supplementary motor area; CC: cingulate cortex. L: left hemisphere; R: right hemisphere.

More activation was observed in a widespread network of bilateral brain areas, including the primary motor cortex, the supplementary motor area, the primary somatosensory cortex, the bilateral dorsolateral prefrontal cortex, the bilateral insula, the inferior frontal gyrus*pars triangularis*and the putamen, when participants avoided sitting stimuli as compared with when participants approached sitting stimuli (Fig. 4).

#### 3.4.3 Avoid sitting > avoid running (HN3)

**Fig. 5.**
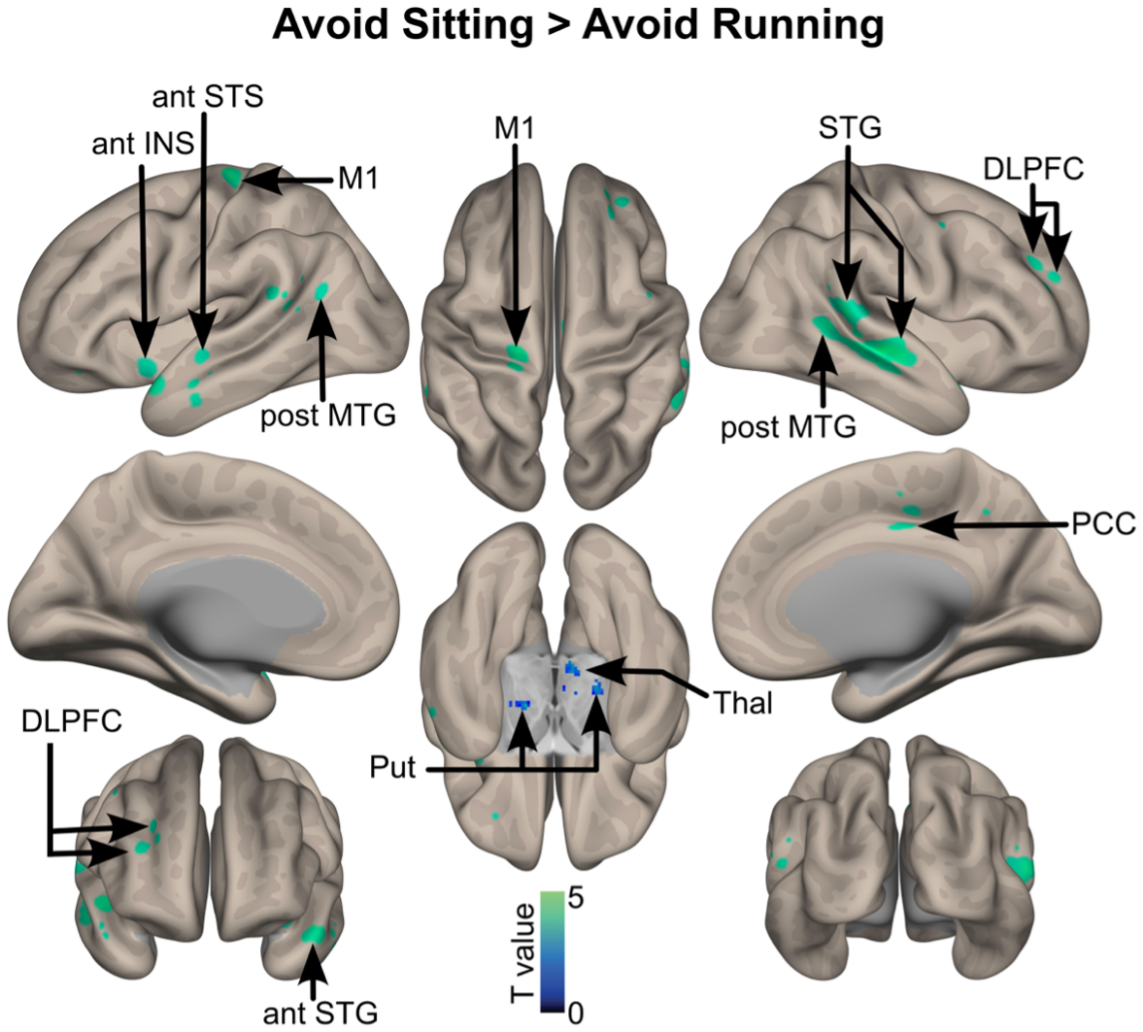
Brain activations when avoiding sedentary stimuli versus approaching physical activity stimuli, corrected for multiple comparisons (whole-brain voxel-wise FDR, q = 0.05, k > 10 voxels). The color bar represents the statistical T value. postSTG: posterior part of the superior temporal gyrus; postMTG: posterior part of the middle temporal gyrus; midSTS: mid part of the superior temporal sulcus; antSTS: anterior part of the superior temporal sulcus; midSTG: mid part of the superior temporal gyrus; M1: primary motor cortex; antINS: insula, anterior part; DLPFC: dorsolateral prefrontal cortex; PCC: posterior cingulate cortex; Thal: thalamus; Put: putamen. L: left hemisphere; R: right hemisphere.

More activation was observed in the left primary motor cortex, insula, anterior superior temporal sulcus, posterior middle temporal gyrus, superior temporal gyrus, posterior cingulate cortex, and dorsolateral prefrontal cortex. Subcortical activations were also observed especially in the bilateral putamen and in the left thalamus, when participants avoided sitting stimuli than when they avoided running stimuli (Fig. 5).

#### 3.4.4 Avoid sitting > avoid standing (HN4)

More activation was observed in the left primary visual cortex, associative visual cortex, temporo-occipital cortex, and superior parietal lobule as well as in the right hemisphere in the similar regions, when participants avoided sitting stimuli as compared with when participants avoided standing stimuli (Supplementary Material 5).

SeeSupplementary Material 6for the detailed coordinates of the clusters presented in this section. Results exploring functional and effective connectivity yielded no significant results.

### 3.5 Associations between subcortical structure shapes and approach–avoidance tendencies

**Fig. 6.**
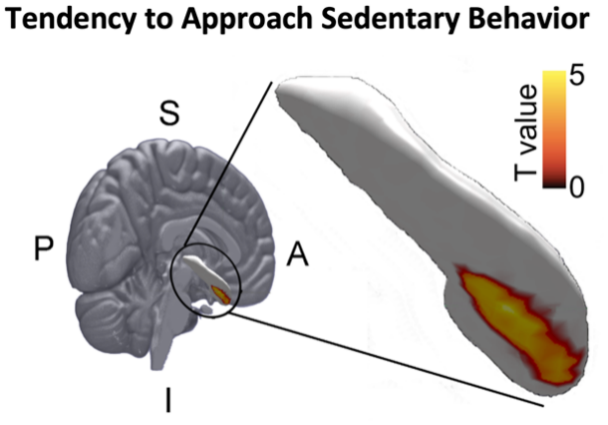
Significant (*p*< 0.05, FWE corrected) positive association between deformation of the right hippocampus and the behavioral tendency to approach sedentary behavior (mean reaction time for approaching sitting < mean reaction time for avoiding sitting). The extent to which approaching sedentary behavior is easier relative to avoiding it is associated with an outward deformation of the inferior/anterior right hippocampus. S: Superior, I: Inferior, P: Posterior, A: Anterior

The association between subcortical structures and the tendency to approach sedentary behavior was assessed by examining the association of errors and reaction times with the extent of deformation of those structures. A greater tendency to approach (vs. avoid) sitting stimuli was associated with larger outward deformations of the right ventral hippocampus (Fig. 6). Errors did not show evidence further supporting this significant association. No other subcortical structure was significantly associated with this tendency. No significant association was observed between approach–avoidance tendencies toward running stimuli and the shape of the subcortical structures.

## 4 Discussion

### 4.1 Main findings

The results of this study, based on an approach–avoidance task during fMRI, showed that avoiding sedentary stimuli (i.e., avatars in a sitting position) is associated with higher levels of behavioral control than avoiding physical activity stimuli (i.e., avatars in a running position). In addition, the outward deformation of the right ventral/anterior hippocampus was associated with a behavioral tendency toward sedentary behavior. These neural results are consistent with behavioral data showing that participants made more errors when avoiding sedentary stimuli than when avoiding physical activity stimuli. Thus, these findings are consistent with TEMPA’s postulate that avoiding sedentary behavior requires more executive control-related processes than approaching sedentary behavior or avoiding physical activity, while they did not provide support for the postulate regarding the rewarding value of sedentary behavior.

### 4.2 Comparison with other studies

#### 4.2.1 Behavioral results

Participants made more errors when avoiding sedentary stimuli than when avoiding physical activity stimuli (HB2). This finding is consistent with the literature that has shown, using a go/no-go task (Duckworth & Kern, 2011), that participants made more commission errors (i.e., a failure to refrain from responding to a “no-go” stimulus) when responding to sedentary stimuli compared with physical activity stimuli (Cheval, Daou, et al., 2020). Thus, these behavioral data provide support for the suggestion that more executive control is required for the avoidance than for approach of sedentary opportunities.

However, our results showed no significant effects of stimulus type, action type, or their interaction on reaction times. This finding contrasts with the literature that has repetitively shown that participants are faster when approaching than when avoiding physical activity stimuli, whereas they are faster when avoiding than when approaching physical inactivity stimuli (Cheval et al., 2014,^2015^;Cheval, Tipura, et al., 2018;Farajzadeh et al., 2023,^2024^;Goubran et al., 2025;Hannan et al., 2019;Moffitt et al., 2019). This discrepancy can be explained by the specificity of the task used in the current study. Specifically, previous studies relied on an explicit approach–avoidance task in which participants were instructed to respond to the content of the image—to approach or avoid depending on the stimulus type (physical activity or sedentary behavior). In contrast, here we used an “implicit” approach–avoidance task in which participants were instructed to respond to the format of the images—to approach or avoid depending on whether the image appeared in portrait or landscape format. A review of the literature found that the implicit stimulus evaluation typically produces smaller effects than explicit stimulus evaluation (Phaf et al., 2014).

Exploratory analyses further revealed that the state of craving for sedentary behavior significantly moderated participants’ reaction times. Specifically, greater craving for sedentary behavior reduced reaction times to sedentary stimuli relative to physical activity stimuli, regardless of the type of action required (i.e., approach or avoidance). These shorter reaction times may be explained by the fact that participants in a state of craving for sedentary behavior may be more attentive to stimuli associated with such behavior. This finding is consistent with previous studies showing that attention is biased toward stimuli that are particularly relevant to participant’s current concerns (Cheval, Miller, et al., 2020;Pool et al., 2016). Accordingly, these findings may suggest that sedentary stimuli may be particularly relevant to the concerns of individuals who self-report a desire to engage in sedentary behavior.

#### 4.2.2 Neural results

fMRI results showed more activation of a motor control network including primary motor cortex, supplementary motor area, primary somatosensory cortex, and dorsolateral prefrontal cortex when participants avoided sedentary stimuli as compared with when participants approached sedentary stimuli. This result suggests that avoiding sedentary behavior may require to deliberately plan and implement the motor action, taking more effort, compared with approach sedentary behavior. However, it is important to note that while this effect was observed specifically for contrasts of sedentary stimuli, the conjunction analysis showed no significant differences across conditions. This calls for caution regarding the specificity of the effect observed for sedentary stimuli. Nevertheless, this suggestion is supported by the larger activation observed in the posterior cingulate cortex and DLPFC when participants avoided sedentary behavior compared with when the avoided physical activity stimuli, which could be related to higher resources required for conflict monitoring as well as action planning and implementation. These observations are consistent with previous EEG studies that have shown, using an approach–avoidance task (Krieglmeyer & Deutsch, 2010) and a go/no-go task (Duckworth & Kern, 2011), that “not going to” or “avoiding” a sedentary stimulus requires greater behavioral control than “not going to” or “avoiding” a physical activity stimulus, as indicated by larger evoked-related potentials in the medial frontal cortex and frontocentral cortex (Cheval et al., 2021;Cheval, Tipura, et al., 2018).

The observed positive association between the outward deformation of the right hippocampus and the tendency to approach (vs. avoid) sedentary behavior was unexpected, as this structure was a priori not associated with motivation or reward-based information processing. To potentially explain these findings, it can be argued that the judgment of stimuli being presented in a portrait or landscape format may have been a confounding factor. The currently perceived function of the hippocampus is to encode spatial and temporal contexts of episodes, constructing a cognitive map (Epstein et al., 2017). In particular, the right hippocampus has been shown to be involved in spatial task performance (Klur et al., 2009). In support of our findings,Hernández et al. (2017)performed an analysis similar to the one presented here, linking cognitive function to hippocampal deformation. They observed that a similar subregion of the right hippocampus was specifically associated with spatial memory performance. To test whether judging the stimulus orientation and/or associated movement acted as a confounder, we performed an additional analysis in which we assessed the association between the reaction time difference between approach versus avoidance of standing stimuli and the structural deformation of the right hippocampus. Such association between hippocampal structure and reaction time to standing stimuli may indicate that the observed effect is driven by the orientation of the stimulus and/or associated movement. This analysis did not show any significant association, providing no evidence that the judgment of the spatial orientation was driving the effect. An alternative explanation may be that the observed associations reflect an emotion-based decision to engage in approach or avoidance behavior. The location of the association with sedentary behavior tendency is mostly ventral/anterior, and this subregion of the hippocampus is associated with the processing of stress, emotion, and affect. Therefore, speculatively, a larger hippocampal capacity to process intrinsically rewarding events may lead to faster responses such as those observed here.

## 5 Limitations and Strengths

This study has several limitations to consider. First, the experimental setup required participants to lie down, which may have influenced their evaluation of the stimuli and reduced ecological validity. Second, the study’s correlational design, without experimental manipulation, limits the ability to establish causal relationships. Third, the use of self-reported measures to assess usual physical activity introduces potential biases and may partially explain the absence of a moderating effect. While we focused on motivational variables as potential moderators of individual differences in task performance, physiological measures, such as VO_2_max, may also play a role. However, these measures are less direct indicators of motivation for physical activity compared with self-reported physical activity levels and craving for physical activity or sedentary behavior. Future research may benefit from incorporating both physiological and motivational indices to better understand how individual differences influence behavioral and neural responses to physical effort. Fourth, while the avatars were validated and relevant to the concept of effort, they cannot fully capture the complexity of effort-related behaviors in real-world contexts. Similarly, while the avatars were designed to maintain consistency in visual complexity and stimulus salience across conditions, in particular by using the same background and ensuring that each avatar appeared in all conditions (i.e., sitting, standing, running), some minor variation is inevitable. These variations should be considered when interpreting differences in reaction times and error rates. Fifth, another potential limitation is related to the 600-ms TR we used, which allowed for better temporal sampling, but may have affected the signal-to-noise ratio and increased the sensitivity to physiological noise (Cordes et al., 2014;Schmitz et al., 2005). To partially address the signal-to-noise-ratio issue, we used an FDR correction, which performs better than FWER in this regard. Future studies should use preprocessing steps such as physiological regressors or Independent Component Analysis to mitigate physiological noise. Despite these limitations, the study has notable strengths. The use of MRI provided high spatial resolution for identifying patterns of brain activations. The design incorporated numerous repetitions within each condition and used a high temporal resolution, optimizing the reliability and quality of the data. The validated stimuli directly addressed the concept of effort, enhancing the study’s relevance, and the well-validated approach–avoidance task added methodological rigor.

## 6 Conclusion

This study provides new insights into the neural mechanisms underlying the difficulty to avoid sedentary stimuli. Behavioral results showed that participants made more errors when avoiding sedentary stimuli than when avoiding physical activity stimuli. Neural results showed greater activation in brain regions associated with motor control, conflict monitoring, and action planning when avoiding sedentary stimuli, suggesting that executive control may play a role in overcoming the tendency to engage in sedentary behavior. These findings align with TEMPA, which posits a natural tendency to minimize effort. More broadly, this study advances our understanding of the factors that shape sedentary and active behaviors and the gap between intentions to be physically active and actual engagement in physical activity. While these findings have potential implications for interventions aimed at promoting physical activity, further research is needed to determine whether targeted training or environmental modifications can help reduce the cognitive demands of overriding sedentary tendencies and support more active lifestyles.

## Supplementary Material

Supplementary Material

## Data Availability

Following good research practices (Boisgontier, 2022), the material, behavioral data, and R scripts are publicly available in Zenodo (Cheval, Ceravolo, Igloi, Zimmermann, et al., 2025).

## References

[IMAG.a.28-b1] Aron , A. R. , Durston , S. , Eagle , D. M. , Logan , G. D. , Stinear , C. M. , & Stuphorn , V. ( 2007 ). Converging evidence for a fronto-basal-ganglia network for inhibitory control of action and cognition . Journal of Neuroscience , 27 ( 44 ), 11860 – 11864 . 10.1523/JNEUROSCI.3644-07.2007 17978025 PMC6673355

[IMAG.a.28-b2] Aron , A. R. , Robbins , T. W. , & Poldrack , R. A. ( 2014 ). Inhibition and the right inferior frontal cortex: One decade on . Trends in Cognitive Sciences , 18 ( 4 ), 177 – 185 . 10.1016/j.tics.2013.12.003 24440116

[IMAG.a.28-b3] Baayen , R. H. , Davidson , D. J. , & Bates , D. M. ( 2008 ). Mixed-effects modeling with crossed random effects for subjects and items . Journal of Memory and Language , 59 ( 4 ), 390 – 412 . 10.1016/j.jml.2007.12.005

[IMAG.a.28-b4] Bargh , J. A. ( 2014 ). The four horsemen of automaticity: Awareness, intention, efficiency, and control in social cognition . In R. S. Wyer , Jr. & T. K. Srull (Eds.), Handbook of social cognition (pp. 1–40). Psychology Press. https://psycnet.apa.org/record/1994-97751-001

[IMAG.a.28-b5] Bates , D. , Mächler , M. , Bolker , B. , & Walker , S. ( 2014 ). Fitting linear mixed-effects models using lme4 . Journal of Statistical Software , 67 ( 1 ), 1 – 48 . 10.18637/jss.v067.i01

[IMAG.a.28-b6] Bennett , C. M. , Wolford , G. L. , & Miller , M. B. ( 2009 ). The principled control of false positives in neuroimaging . Social Cognitive and Affective Neuroscience , 4 ( 4 ), 417 – 422 . 10.1093/scan/nsp053 20042432 PMC2799957

[IMAG.a.28-b7] Boisgontier , M. ( 2022 ). Research integrity requires to be aware of good and questionable research practices . European Rehabilitation Journal , 2 ( 1 ), 1 – 3 . 10.52057/erj.v2i1.24

[IMAG.a.28-b8] Boisgontier , M. P. , & Cheval , B. ( 2016 ). The anova to mixed model transition . Neuroscience & Biobehavioral Reviews , 68 , 1004 – 1005 . 10.1016/j.neubiorev.2016.05.034 27241200

[IMAG.a.28-b9] Boisgontier , M. P. , Cheval , B. , Chalavi , S. , van Ruitenbeek , P. , Leunissen , I. , Levin , O. , Nieuwboer , A. , & Swinnen , S. P. ( 2016 ). Individual differences in brainstem and basal ganglia structure predict postural control and balance loss in young and older adults . Neurobiology of Aging , 50 , 47 – 59 . 10.1016/j.neurobiolaging.2016.10.024 27875755

[IMAG.a.28-b10] Boisgontier , M. P. , van Ruitenbeek , P. , Leunissen , I. , Chalavi , S. , Sunaert , S. , Levin , O. , & Swinnen , S. P. ( 2016 ). Nucleus accumbens and caudate atrophy predicts longer action selection times in young and old adults . Human Brain Mapping , 37 ( 12 ), 4629 – 4639 . 10.1002/hbm.23333 27585251 PMC6867567

[IMAG.a.28-b11] Brand , R. , & Ekkekakis , P. ( 2018 ). Affective–Reflective Theory of physical inactivity and exercise . German Journal of Exercise and Sport Research , 48 ( 1 ), 48 – 58 . 10.1007/s12662-017-0477-9

[IMAG.a.28-b12] Burgaleta , M. , MacDonald , P. A. , Martínez , K. , Roman , F. J. , Álvarez‐Linera , J. , Gonzalez , A. R. , Karama , S. , & Colom , R. ( 2014 ). Subcortical regional morphology correlates with fluid and spatial intelligence . Human Brain Mapping , 35 ( 5 ), 1957 – 1968 . 10.1002/hbm.22305 23913782 PMC6869737

[IMAG.a.28-b13] Cheval , B. , Bacelar , M. , Daou , M. , Cabral , A. , Parma , J. , Forestier , C. , Orsholits , D. , Sander , D. , Boisgontier , M. , & Miller , M. W. ( 2020 ). Higher inhibitory control is required to escape the innate attraction to effort minimization . Psychology of Sport and Exercise , 51 , 101781 . 10.1016/j.psychsport.2020.101781

[IMAG.a.28-b14] Cheval , B. , Boisgontier , M. , Sieber , S. , Ihle , A. , Orsholits , D. , Forestier , C. , Sander , D. , & Chalabaev , A. ( 2022 ). Cognitive functions and physical activity in aging when energy is lacking . European Journal of Ageing , 19 , 533 – 544 . 10.1007/s10433-021-00654-2 36052203 PMC9424387

[IMAG.a.28-b15] Cheval , B. , & Boisgontier , M. P. ( 2021 ). The theory of effort minimization in physical activity . Exercise and Sport Sciences Reviews , 49 ( 3 ), 168 – 178 . 10.1249/JES.0000000000000252 34112744 PMC8191473

[IMAG.a.28-b16] Cheval , B. , & Boisgontier , M. P. ( 2024 ). Promouvoir une activité physique régulière chez les patients: L’importance de la perception de l’effort . STAPS , 146 ( 3 ), 115 – 134 . 10.3917/sta.146.0115

[IMAG.a.28-b17] Cheval , B. , Boisgontier , M. P. , Bacelar , M. F. , Feiss , R. , & Miller , M. W. ( 2019 ). Opportunities to sit and stand trigger equivalent reward-related brain activity . International Journal of Psychophysiology , 141 , 9 – 17 . 10.1016/j.ijpsycho.2019.04.009 31029733

[IMAG.a.28-b18] Cheval , B. , Cabral , D. A. R. , Daou , M. , Bacelar , M. , Parma , J. O. , Forestier , C. , Orsholits , D. , Maltagliati , S. , Sander , D. , & Boisgontier , M. P. ( 2021 ). Inhibitory control elicited by physical activity and inactivity stimuli: An EEG study . Motivation Science , 7 ( 4 ), 386 – 389 . 10.1037/mot0000236

[IMAG.a.28-b19] Cheval , B. , Ceravolo , L. , Igloi , K. , Sander , D. , Zimmermann , M. , Van Ruitenbeek , P. , & Boisgontier , M. P. ( 2025 ). Neural correlates of approach and avoidance tendencies toward physical activity and sedentary stimuli: An fMRI study . bioRxiv . 10.1101/2025.01.10.632302 PMC12319747 40800852

[IMAG.a.28-b20] Cheval , B. , Ceravolo , L. , Igloi , K. , Zimmermann , M. , van Ruitenbeek , P. , & Boisgontier , M. P. ( 2025 ). Neural correlates of approach and avoidance tendencies toward physical activity and sedentary stimuli: An fMRI study . [Dataset]. 10.5281/zenodo.14636750 . PMC12319747 40800852

[IMAG.a.28-b21] Cheval , B. , Daou , M. , Cabral , D. A. R. , Bacelar , M. , Parma , J. O. , Forestier , C. , Orsholits , D. , Sander , D. , Boisgontier , M. P. , & Miller , M. W. ( 2020 ). Higher inhibitory control is required to escape the innate attraction to effort minimization . Psychology of Sport and Exercise , 51 , 101781 . 10.1016/j.psychsport.2020.101781

[IMAG.a.28-b22] Cheval , B. , Maltagliati , S. , Fessler , L. , Farajzadeh , A. , Abdallah , S. N. B. , Vogt , F. , Dubessy , M. , Lacour , M. , Miller , M. W. , & Sander , D. ( 2022 ). Physical effort biases the perceived pleasantness of neutral faces: A virtual reality study . Psychology of Sport and Exercise , 63 , 102287 . 10.1016/j.psychsport.2022.102287

[IMAG.a.28-b23] Cheval , B. , Miller , M. W. , Orsholits , D. , Berry , T. , Sander , D. , & Boisgontier , M. P. ( 2020 ). Physically active individuals look for more: An eye-tracking study of attentional bias . Psychophysiology , 57 ( 6 ), e13582 . 10.1111/psyp.13582 32277857

[IMAG.a.28-b24] Cheval , B. , Orsholits , D. , Sieber , S. , Courvoisier , D. C. , Cullati , S. , & Boisgontier , M. P. ( 2020 ). Relationship between decline in cognitive resources and physical activity . Health Psychology , 39 ( 6 ), 519 – 528 . 10.1037/hea0000857 32202828

[IMAG.a.28-b25] Cheval , B. , Radel , R. , Neva , J. L. , Boyd , L. A. , Swinnen , S. P. , Sander , D. , & Boisgontier , M. P. ( 2018 ). Behavioral and neural evidence of the rewarding value of exercise behaviors: A systematic review . Sports Medicine , 48 ( 6 ), 1389 – 1404 . 10.1007/s40279-018-0898-0 29556981

[IMAG.a.28-b26] Cheval , B. , Rebar , A. L. , Miller , M. M. , Sieber , S. , Orsholits , D. , Baranyi , G. , Courvoisier , D. C. , Cullati , S. , Sander , D. , & Boisgontier , M. P. ( 2019 ). Cognitive resources moderate the adverse impact of poor neighborhood conditions on physical activity . Preventive Medicine , 126 , 105741 . 10.1016/j.ypmed.2019.05.029 31153916

[IMAG.a.28-b27] Cheval , B. , Saoudi , I. , Maltagliati , S. , Fessler , L. , Farajzadeh , A. , Sieber , S. , Cullati , S. , & Boisgontier , M. ( 2023 ). Initial status and change in cognitive function mediate the association between academic education and physical activity in adults over 50 years of age . Psychology and Aging , 38 ( 6 ), 494 – 507 . 10.1037/pag0000749 37166860

[IMAG.a.28-b28] Cheval , B. , Sarrazin , P. , Isoard-Gautheur , S. , Radel , R. , & Friese , M. ( 2015 ). Reflective and impulsive processes explain (in)effectiveness of messages promoting physical activity: A randomized controlled trial . Health Psychology , 34 ( 1 ), 10 – 19 . 10.1037/hea0000102 25133840

[IMAG.a.28-b29] Cheval , B. , Sarrazin , P. , & Pelletier , L. ( 2014 ). Impulsive approach tendencies towards physical activity and sedentary behaviors, but not reflective intentions, prospectively predict non-exercise activity thermogenesis . PLoS One , 9 ( 12 ), e115238 . 10.1371/journal.pone.0115238 25526596 PMC4272300

[IMAG.a.28-b30] Cheval , B. , Tipura , E. , Burra , N. , Frossard , J. , Chanal , J. , Orsholits , D. , Radel , R. , & Boisgontier , M. P. ( 2018 ). Avoiding sedentary behaviors requires more cortical resources than avoiding physical activity: An EEG study . Neuropsychologia , 119 , 68 – 80 . 10.1016/j.neuropsychologia.2018.07.029 30056055

[IMAG.a.28-b31] Cheval , B. , Zou , L. , Maltagliati , S. , Fessler , L. , Owen , N. , Falck , R. S. , Yu , Q. , Zhang , Z. , & Dupuy , O. ( 2024 ). Intention–behaviour gap in physical activity: Unravelling the critical role of the automatic tendency towards effort minimisation . British Journal of Sports Medicine , 58 ( 16 ), 889 – 891 . 10.1136/bjsports-2024-108144 38777385

[IMAG.a.28-b32] Collins , D. L. , Neelin , P. , Peters , T. M. , & Evans , A. C. ( 1994 ). Automatic 3D intersubject registration of MR volumetric data in standardized Talairach space . Journal of Computer Assisted Tomography , 18 ( 2 ), 192 – 205 . https://pubmed.ncbi.nlm.nih.gov/8126267/ 8126267

[IMAG.a.28-b33] Conroy , D. E. , & Berry , T. R. ( 2017 ). Automatic affective evaluations of physical activity . Exercise and Sport Sciences Reviews , 45 ( 4 ), 230 – 237 . 10.1249/JES.0000000000000120 28704217

[IMAG.a.28-b34] Corbit , L. H. , & Balleine , B. W. ( 2011 ). The general and outcome-specific forms of Pavlovian-instrumental transfer are differentially mediated by the nucleus accumbens core and shell . Journal of Neuroscience , 31 ( 33 ), 11786 – 11794 . 10.1523/JNEUROSCI.2711-11.2011 21849539 PMC3208020

[IMAG.a.28-b35] Cordes , D. , Nandy , R. R. , Schafer , S. , & Wager , T. D. ( 2014 ). Characterization and reduction of cardiac-and respiratory-induced noise as a function of the sampling rate (TR) in fMRI . NeuroImage , 89 , 314 – 330 . 10.1016/j.neuroimage.2013.12.013 24355483 PMC4209749

[IMAG.a.28-b36] Craig , C. L. , Marshall , A. L. , Sjostrom , M. , Bauman , A. E. , Booth , M. L. , Ainsworth , B. E. , Pratt , M. , Ekelund , U. , Yngve , A. , Sallis , J. F. , & Oja , P. ( 2003 ). International physical activity questionnaire: 12-country reliability and validity . Medicine and Science in Sports and Exercise , 35 ( 8 ), 1381 – 1395 . 10.1249/01.MSS.0000078924.61453.FB 12900694

[IMAG.a.28-b37] Crémers , J. , Dessoullières , A. , & Garraux , G. ( 2012 ). Hemispheric specialization during mental imagery of brisk walking . Human Brain Mapping , 33 ( 4 ), 873 – 882 . 10.1002/hbm.21255 21425400 PMC6870080

[IMAG.a.28-b38] Csajbók , Z. , Sieber , S. , Cullati , S. , Cermakova , P. , & Cheval , B. ( 2022 ). Physical activity partly mediates the association between cognitive function and depressive symptoms . Translational Psychiatry , 12 ( 1 ), 414 . 10.1038/s41398-022-02191-7 36167692 PMC9515096

[IMAG.a.28-b39] Daly , M. , McMinn , D. , & Allan , J. L. ( 2015 ). A bidirectional relationship between physical activity and executive function in older adults . Frontiers in Human Neuroscience , 8 , 1044 . 10.3389/fnhum.2014.01044 25628552 PMC4292779

[IMAG.a.28-b40] Davis , C. , Katzman , D. K. , Kaptein , S. , Kirsh , C. , Brewer , H. , Kalmbach , K. , Olmsted , M. F. , Woodside , D. B. , & Kaplan , A. S. ( 1997 ). The prevalence of high-level exercise in the eating disorders: Etiological implications . Comprehensive Psychiatry , 38 ( 6 ), 321 – 326 . 10.1016/S0010-440X(97)90927-5 9406737

[IMAG.a.28-b41] Duckworth , A. L. , & Kern , M. L. ( 2011 ). A meta-analysis of the convergent validity of self-control measures . Journal of Research in Personality , 45 ( 3 ), 259 – 268 . 10.1016/j.jrp.2011.02.004 21643479 PMC3105910

[IMAG.a.28-b42] Elliot , A. J. , & Thrash , T. M. ( 2010 ). Approach and avoidance temperament as basic dimensions of personality . Journal of Personality , 78 ( 3 ), 865 – 906 . 10.1111/j.1467-6494.2010.00636.x 20573129

[IMAG.a.28-b43] Epstein , R. A. , Patai , E. Z. , Julian , J. B. , & Spiers , H. J. ( 2017 ). The cognitive map in humans: Spatial navigation and beyond . Nature Neuroscience , 20 ( 11 ), 1504 – 1513 . 10.1038/nn.4656 29073650 PMC6028313

[IMAG.a.28-b44] Farajzadeh , A. , Goubran , M. , Beehler , A. , Cherkawi , N. , Morrison , P. , de Chanaleilles , M., Maltagliati , S. , Cheval , B. , Miller , M. W. , Sheehy , L. , & Boisgontier , M. ( 2023 ). Automatic approach-avoidance tendency toward physical activity, sedentary, and neutral stimuli as a function of age, explicit affective attitude, and intention to be active . Peer Community Journal , 3 , e21 . 10.24072/pcjournal.246 PMC7617180 39659553

[IMAG.a.28-b45] Farajzadeh , A. , Jabouille , F. , Benoit , N. , Bezeau , O. , Bourgie , T. , Gerro , B. , Ouimet , J. , & Boisgontier , M. P. ( 2024 ). Apathy, intentions, explicit attitudes, and approach-avoidance tendencies in physical activity behavior . Communications in Kinesiology , 1 ( 6 ). 10.1101/2024.07.16.24310493

[IMAG.a.28-b46] Faul , F. , Erdfelder , E. , Buchner , A. , & Lang , A.-G. ( 2009 ). Statistical power analyses using G* Power 3.1: Tests for correlation and regression analyses . Behavior Research Methods , 41 ( 4 ), 1149 – 1160 . 10.3758/BRM.41.4.1149 19897823

[IMAG.a.28-b47] Frossard , J. , & Renaud , O. ( 2019 ) . The correlation structure of mixed effects models with crossed random effects in controlled experiments . https://arxiv.org/abs/1903.10766 .

[IMAG.a.28-b48] Gottfried , J. A. , O’Doherty , J. , & Dolan , R. J. ( 2003 ). Encoding predictive reward value in human amygdala and orbitofrontal cortex . Science , 301 ( 5636 ), 1104 – 1107 . 10.1126/science.1087919 12934011

[IMAG.a.28-b49] Goubran , M. , Zammar , C. , Tellez Alvarez , S., Heran , E. , Proulx , S. , Bilodeau , M. , & Boisgontier , M. P. ( 2025 ). Approach-avoidance tendencies influence the relationship between fear of movement and physical activity in osteoarthritis . MedRxiv . 10.1101/2025.01.23.25321044

[IMAG.a.28-b50] Griffiths , M. , Szabo , A. , & Terry , A. ( 2005 ). The exercise addiction inventory: A quick and easy screening tool for health practitioners . British Journal of Sports Medicine , 39 ( 6 ), e30 – e30 . 10.1136/bjsm.2004.017020 15911594 PMC1725234

[IMAG.a.28-b51] Hannan , T. E. , Moffitt , R. L. , Neumann , D. L. , & Kemps , E. ( 2019 ). Implicit approach–avoidance associations predict leisure-time exercise independently of explicit exercise motivation . Sport, Exercise, and Performance Psychology , 8 ( 2 ), 210 – 222 . 10.1037/spy0000145

[IMAG.a.28-b52] Hernández , M. d. C. V. , Cox , S. R. , Kim , J. , Royle , N. A. , Maniega , S. M. , Gow , A. J. , Anblagan , D. , Bastin , M. E. , Park , J. , & Starr , J. M. ( 2017 ). Hippocampal morphology and cognitive functions in community-dwelling older people: The Lothian Birth Cohort 1936 . Neurobiology of Aging , 52 , 1 – 11 . 10.1016/j.neurobiolaging.2016.12.012 28104542 PMC5364373

[IMAG.a.28-b53] Jackson , T. , Gao , X. , & Chen , H. ( 2014 ). Differences in neural activation to depictions of physical exercise and sedentary activity: An fMRI study of overweight and lean Chinese women . International Journal of Obesity , 38 ( 9 ), 1180 . 10.1038/ijo.2013.245 24366575

[IMAG.a.28-b54] Jacobsen , L. K. , Gore , J. C. , Skudlarski , P. , Lacadie , C. M. , Jatlow , P. , & Krystal , J. H. ( 2002 ). Impact of intravenous nicotine on BOLD signal response to photic stimulation . Magnetic Resonance Imaging , 20 ( 2 ), 141 – 145 . 10.1016/S0730-725X(02)00494-0 12034334

[IMAG.a.28-b55] Jenkinson , M. , Beckmann , C. F. , Behrens , T. E. , Woolrich , M. W. , & Smith , S. M. ( 2012 ). FSL . NeuroImage , 62 ( 2 ), 782 – 790 . 10.1016/j.neuroimage.2011.09.015 21979382

[IMAG.a.28-b56] Klur , S. , Muller , C. , Pereira de Vasconcelos , A. , Ballard , T. , Lopez , J. , Galani , R. , Certa , U. , & Cassel , J. C. ( 2009 ). Hippocampal‐dependent spatial memory functions might be lateralized in rats: An approach combining gene expression profiling and reversible inactivation . Hippocampus , 19 ( 9 ), 800 – 816 . 10.1002/hipo.20562 19235229

[IMAG.a.28-b57] Knutson , B. , Adams , C. M. , Fong , G. W. , & Hommer , D. ( 2001 ). Anticipation of increasing monetary reward selectively recruits nucleus accumbens . Journal of Neuroscience , 21 ( 16 ), RC159 . 10.1523/JNEUROSCI.21-16-j0002.2001 11459880 PMC6763187

[IMAG.a.28-b58] Krieglmeyer , R. , & Deutsch , R. ( 2010 ). Comparing measures of approach–avoidance behaviour: The manikin task vs. two versions of the joystick task . Cognition and Emotion , 24 ( 5 ), 810 – 828 . 10.1080/02699930903047298

[IMAG.a.28-b59] Kullmann , S. , Giel , K. E. , Hu , X. , Bischoff , S. C. , Teufel , M. , Thiel , A. , Zipfel , S. , & Preissl , H. ( 2014 ). Impaired inhibitory control in anorexia nervosa elicited by physical activity stimuli . Social Cognitive and Affective Neuroscience , 9 ( 7 ), 917 – 923 . 10.1093/scan/nst070 23677490 PMC4090959

[IMAG.a.28-b60] Kuznetsova , A. , Brockhoff , P. B. , & Christensen , R. H. B. ( 2015 ). lmerTest Package: Tests in linear mixed effects models . Journal of Statistical Software , 82 ( 13 ). 10.18637/jss.v082.i13

[IMAG.a.28-b61] Lindquist , M. A. , & Mejia , A. ( 2015 ). Zen and the art of multiple comparisons . Psychosomatic Medicine , 77 ( 2 ), 114 – 125 . 10.1097/PSY.0000000000000148 25647751 PMC4333023

[IMAG.a.28-b62] Maltagliati , S. , Fessler , L. , Yu , Q. , Zhang , Z. , Chen , Y. , Dupuy , O. , Falck , R. , Owen , N. , Zou , L. , & Cheval , B. ( 2024 ). Effort minimization: A permanent, dynamic, and surmountable influence on physical activity . Journal of Sport and Health Science , 14 , 100971 . 10.1016/j.jshs.2024.100971 39233203 PMC11809139

[IMAG.a.28-b63] Maltagliati , S. , Raichlen , D. A. , Rhodes , R. E. , & Cheval , B. ( 2025 ). Closing the intention-behavior gap in physical activity: The moderating effect of individual differences in the valuation of physical effort . British Journal of Health Psychology , 30 ( 2 ), e12790 . 10.1111/bjhp.12790 40130726 PMC13729771

[IMAG.a.28-b64] McDermott , T. J. , Kirlic , N. , Akeman , E. , Touthang , J. , Clausen , A. N. , Kuplicki , R. , & Aupperle , R. L. ( 2021 ). Test–retest reliability of approach‐avoidance conflict decision‐making during functional magnetic resonance imaging in healthy adults . Human Brain Mapping , 42 ( 8 ), 2347 – 2361 . 10.1002/hbm.25371 33650761 PMC8090786

[IMAG.a.28-b65] Moffitt , R. L. , Kemps , E. , Hannan , T. E. , Neumann , D. L. , Stopar , S. P. , & Anderson , C. J. ( 2019 ). Implicit approach biases for physically active lifestyle cues . International Journal of Sport and Exercise Psychology , 18 ( 6 ), 833 – 849 . 10.1080/1612197X.2019.1581829

[IMAG.a.28-b66] Nash , J. C. , & Varadhan , R. ( 2011 ). Unifying optimization algorithms to aid software system users: Optimx for R . Journal of Statistical Software , *43* (9). 10.18637/jss.v043.i09

[IMAG.a.28-b67] Nelder , J. A. , & Mead , R. ( 1965 ). A simplex method for function minimization . The Computer Journal , 7 ( 4 ), 308 – 313 . 10.1093/comjnl/7.4.308

[IMAG.a.28-b68] Oldfield , R. C. ( 1971 ). The assessment and analysis of handedness: The Edinburgh inventory . Neuropsychologia , 9 ( 1 ), 97 – 113 . 10.1016/0028-3932(71)90067-4 5146491

[IMAG.a.28-b69] Parma , J. , Bacelar , M. , Cabral , D. , Recker , R. , Renaud , O. , Sander , D. , Krigolson , O. , Miller , M. , Cheval , B. , & Boisgontier , M. ( 2023 ). Relationship between reward-related brain activity and opportunities to sit . Cortex , 167 , 197 – 217 . 10.1016/j.cortex.2023.06.011 37572531

[IMAG.a.28-b70] Patenaude , B. , Smith , S. M. , Kennedy , D. N. , & Jenkinson , M. ( 2011 ). A Bayesian model of shape and appearance for subcortical brain segmentation . NeuroImage , 56 ( 3 ), 907 – 922 . 10.1016/j.neuroimage.2011.02.046 21352927 PMC3417233

[IMAG.a.28-b71] Phaf , R. H. , Mohr , S. E. , Rotteveel , M. , & Wicherts , J. M. ( 2014 ). Approach, avoidance, and affect: A meta-analysis of approach-avoidance tendencies in manual reaction time tasks . Frontiers in Psychology , 5 , 378 . 10.3389/fpsyg.2014.00378 24847292 PMC4021119

[IMAG.a.28-b72] Pool , E. , Brosch , T. , Delplanque , S. , & Sander , D. ( 2016 ). Attentional bias for positive emotional stimuli: A meta-analytic investigation . Psychological Bulletin , 142 , 79 – 106 . 10.1037/bul0000026 26390266

[IMAG.a.28-b73] Powell , M. J. ( 2009 ). The BOBYQA algorithm for bound constrained optimization without derivatives . Cambridge NA Report NA2009/06, University of Cambridge, Cambridge , 26 – 46 . https://www.damtp.cam.ac.uk/user/na/NA_papers/NA2009_06.pdf

[IMAG.a.28-b74] Prévost , C. , Liljeholm , M. , Tyszka , J. M. , & O’Doherty , J. P. ( 2012 ). Neural correlates of specific and general Pavlovian-to-Instrumental Transfer within human amygdalar subregions: A high-resolution fMRI study . Journal of Neuroscience , 32 ( 24 ), 8383 – 8390 . 10.1523/JNEUROSCI.6237-11.2012 22699918 PMC6703659

[IMAG.a.28-b75] Prévost , C. , Pessiglione , M. , Météreau , E. , Cléry-Melin , M.-L. , & Dreher , J.-C. ( 2010 ). Separate valuation subsystems for delay and effort decision costs . Journal of Neuroscience , 30 ( 42 ), 14080 – 14090 . 10.1523/JNEUROSCI.2752-10.2010 20962229 PMC6634773

[IMAG.a.28-b76] Pruessner , J. C. , Li , L. M. , Serles , W. , Pruessner , M. , Collins , D. L. , Kabani , N. , Lupien , S. , & Evans , A. C. ( 2000 ). Volumetry of hippocampus and amygdala with high-resolution MRI and three-dimensional analysis software: Minimizing the discrepancies between laboratories . Cerebral Cortex , 10 ( 4 ), 433 – 442 . 10.1093/cercor/10.4.433 10769253

[IMAG.a.28-b77] R Core Team . ( 2019 ). R: A language and environment for statistical computing . Vienna, Austria. https://www.R-project.org/

[IMAG.a.28-b78] Roesch , M. R. , & Olson , C. R. ( 2004 ). Neuronal activity related to reward value and motivation in primate frontal cortex . Science , 304 ( 5668 ), 307 – 310 . 10.1126/science.1093223 15073380

[IMAG.a.28-b79] Rolls , E. T. , Huang , C.-C. , Lin , C.-P. , Feng , J. , & Joliot , M. ( 2020 ). Automated anatomical labelling atlas 3 . NeuroImage , 206 , 116189 . 10.1016/j.neuroimage.2019.116189 31521825

[IMAG.a.28-b80] Rougier , M. , Muller , D. , Ric , F. , Alexopoulos , T. , Batailler , C. , Smeding , A. , & Aubé , B. ( 2018 ). A new look at sensorimotor aspects in approach/avoidance tendencies: The role of visual whole-body movement information . Journal of Experimental Social Psychology , 76 , 42 – 53 . 10.1016/j.jesp.2017.12.004

[IMAG.a.28-b81] Sabia , S. , Dugravot , A. , Dartigues , J.-F. , Abell , J. , Elbaz , A. , Kivimäki , M. , & Singh-Manoux , A. ( 2017 ). Physical activity, cognitive decline, and risk of dementia: 28 year follow-up of Whitehall II cohort study . British Medical Journal , 357 , j2709 . 10.1136/bmj.j2709 28642251 PMC5480222

[IMAG.a.28-b82] Schmitz , B. L. , Aschoff , A. J. , Hoffmann , M. H. , & Grön , G. ( 2005 ). Advantages and pitfalls in 3T MR brain imaging: A pictorial review . American Journal of Neuroradiology , 26 ( 9 ), 2229 – 2237 . http://www.ajnr.org/content/26/9/2229 16219827 PMC7976112

[IMAG.a.28-b83] Schultz , W. , Tremblay , L. , & Hollerman , J. R. ( 2000 ). Reward processing in primate orbitofrontal cortex and basal ganglia . Cerebral Cortex , 10 ( 3 ), 272 – 283 . 10.1093/cercor/10.3.272 10731222

[IMAG.a.28-b84] Smith , S. M. , Jenkinson , M. , Woolrich , M. W. , Beckmann , C. F. , Behrens , T. E. , Johansen-Berg , H. , Bannister , P. R. , De Luca , M., Drobnjak , I. , & Flitney , D. E. ( 2004 ). Advances in functional and structural MR image analysis and implementation as FSL . NeuroImage , 23 , S208 – S219 . 10.1016/j.neuroimage.2004.07.051 15501092

[IMAG.a.28-b85] Smith , S. M. , & Nichols , T. E. ( 2009 ). Threshold-free cluster enhancement: Addressing problems of smoothing, threshold dependence and localisation in cluster inference . NeuroImage , 44 ( 1 ), 83 – 98 . 10.1016/j.neuroimage.2008.03.061 18501637

[IMAG.a.28-b86] Strack , F. , & Deutsch , R. ( 2004 ). Reflective and impulsive determinants of social behavior . Personality and Social Psychology Review , 8 ( 3 ), 220 – 247 . 10.1207/s15327957pspr0803_1 15454347

[IMAG.a.28-b87] Whitfield-Gabrieli , S. , & Nieto-Castanon , A. ( 2012 ). Conn: A functional connectivity toolbox for correlated and anticorrelated brain networks . Brain Connectivity , 2 ( 3 ), 125 – 141 . 10.1089/brain.2012.0073 22642651

[IMAG.a.28-b88] Woolrich , M. W. , Jbabdi , S. , Patenaude , B. , Chappell , M. , Makni , S. , Behrens , T. , Beckmann , C. , Jenkinson , M. , & Smith , S. M. ( 2009 ). Bayesian analysis of neuroimaging data in FSL . NeuroImage , 45 ( 1 ), S173 – S186 . 10.1016/j.neuroimage.2008.10.055 19059349

[IMAG.a.28-b89] Xu , C. , Xu , H. , Yang , Z. , & Guo , C. ( 2023 ). Regional shape alteration of left thalamus associated with late chronotype in young adults . Chronobiology International , 40 ( 3 ), 234 – 245 . 10.1080/07420528.2022.2162916 36597182

[IMAG.a.28-b90] Zandbelt , B. B. , & Vink , M. ( 2010 ). On the role of the striatum in response inhibition . PLoS One , 5 ( 11 ), e13848 . 10.1371/journal.pone.0013848 21079814 PMC2973972

