# Supplementary material for: Neural correlates of approach–avoidance tendencies toward physical activity and sedentary stimuli: An MRI study
Source: Imaging Neurosci (Camb). 2025 Jun 6;3:IMAG.a.28. doi: 10.1162/IMAG.a.28 (PMC12319747; doi:10.1162/IMAG.a.28)
Supplement: Supplementary Material [file imag.a.28_supp.pdf]

SUPPLEMENTARY MATERIAL

Neural correlates of approach-avoidance tendencies toward physical activity and sedentary stimuli: An MRI study

Boris Cheval, Leonardo Ceravolo, Ophelia Zimmermann, Kinga Igloi, David Sander, Peter van Ruitenbeek, Matthieu P. Boisgontier

Supplementary Material 1. Sample of stimuli used in each condition

Supplementary Material 2. Measures of the dispositional and situational moderators

Supplementary Material 3. Results of the exploratory analyses for reaction times

Supplementary Material 4. Results of the exploratory analyses for errors

Supplementary Material 5. Neural activity associated with the avoidance of sitting stimuli: Avoid Sitting > Avoid Standing

Supplementary Material 6. Detailed coordinates of the clusters presented in this section

Supplementary Material 1. Sample of stimuli used in each condition

Running condition

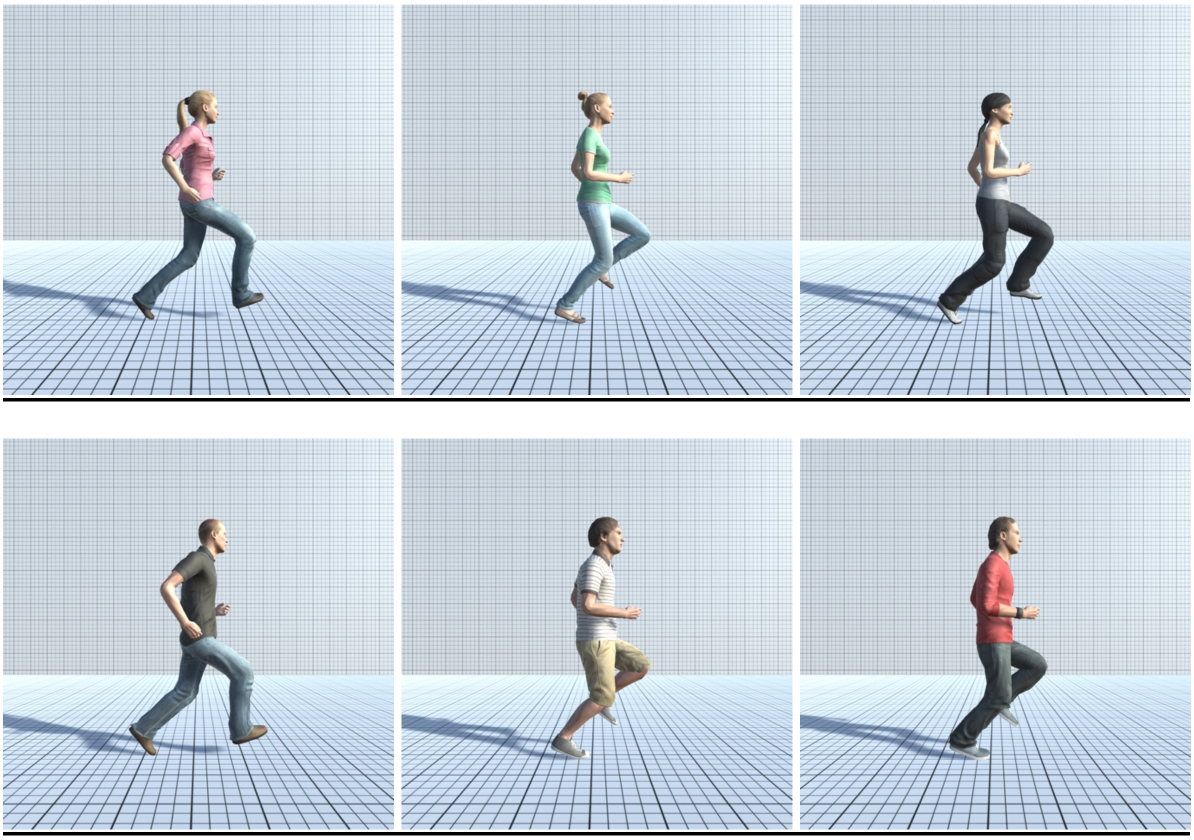

Sitting condition

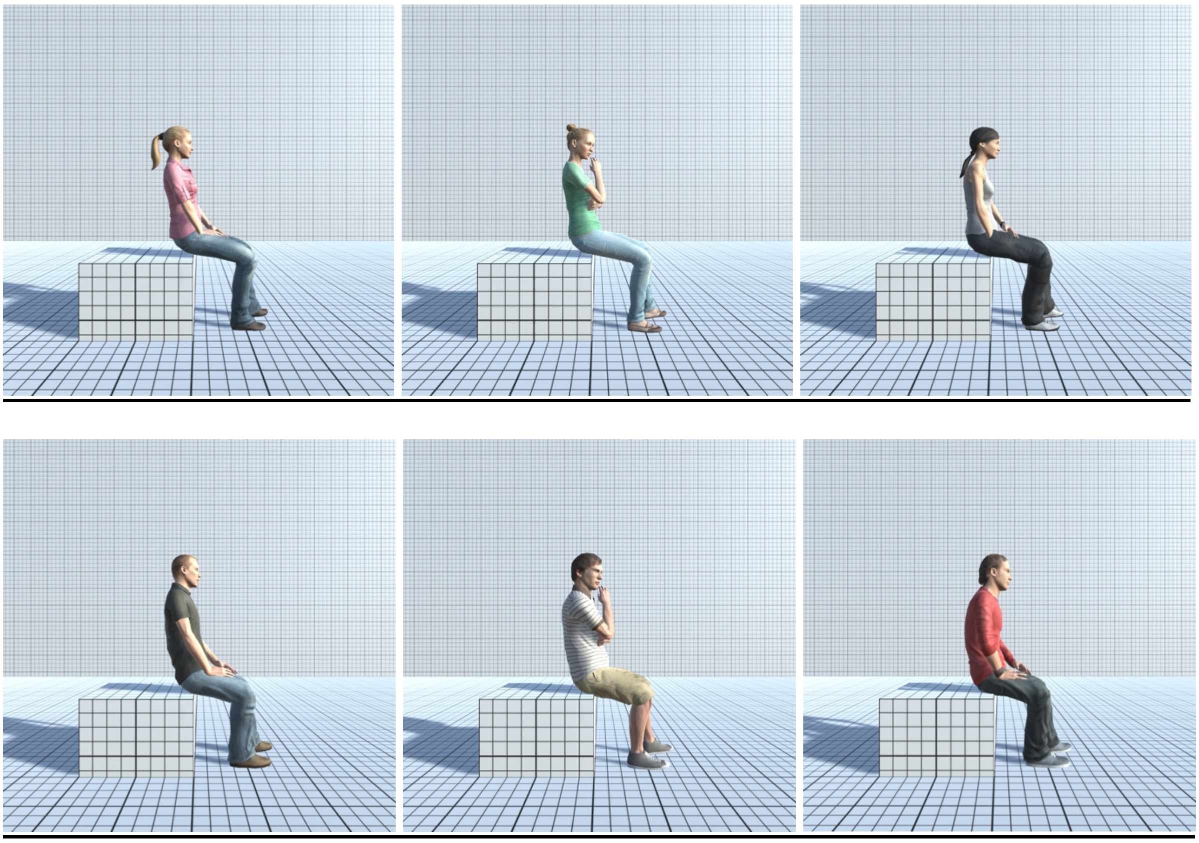

Standing condition

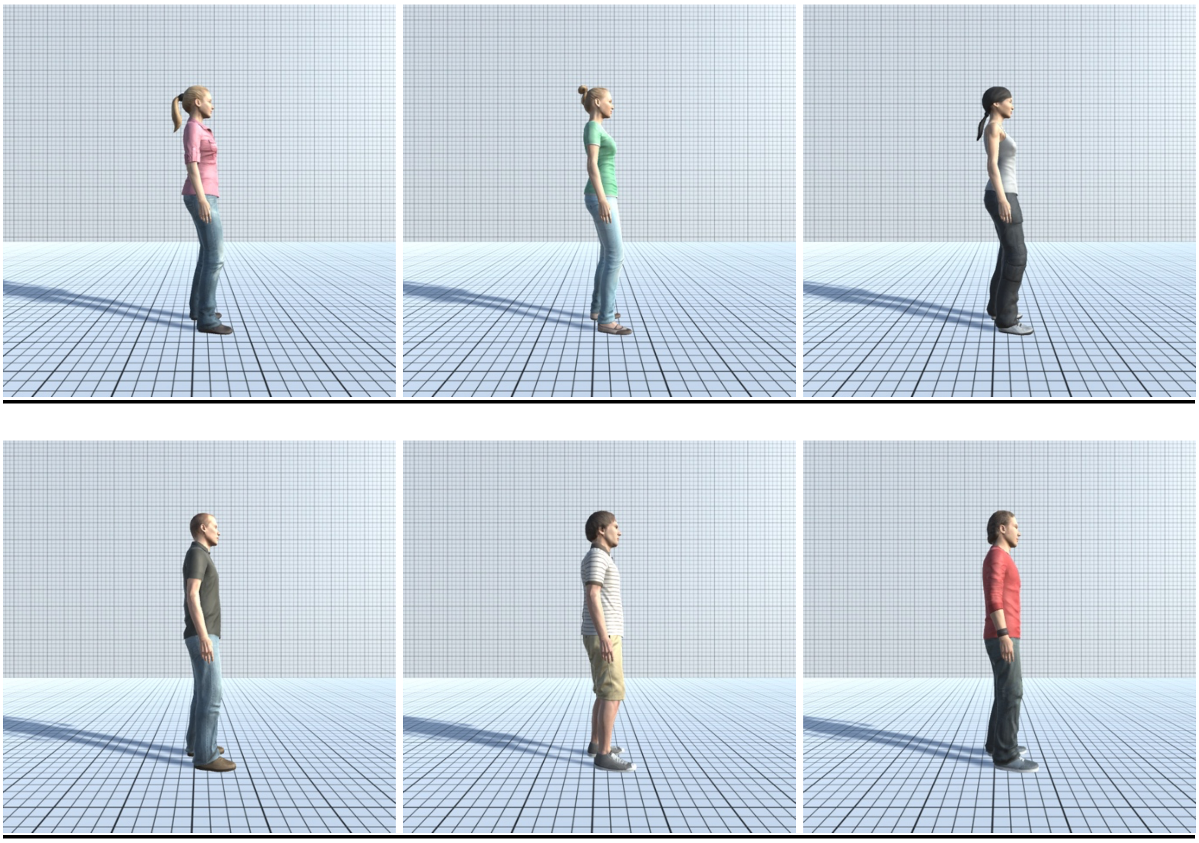

Supplementary Material 2. Measures of the dispositional and situational moderators

Usual level of physical activity

Commuting and leisure time physical activity was assessed using an adapted version of the International Physical Activity Questionnaire. Specifically, the first set of questions focused on the sports-related physical activity, the second on commuter-related physical activity (i.e., walking and cycling), and the last set of questions focused on leisure-time related physical activity (i.e., walking, moderate physical activity, vigorous physical activity). Here we created a score that is the sum of all the time spent in these different physical activities.

Craving for sedentary behaviors

Sedentary craving was assessed with the following item: "At this moment, to what extent do you feel the desire (urge) to engage in sedentary activities (e.g., sitting, resting, watching TV)?". Participants respond on a scale from 1 "no desire at all" to 7 "very strong desire".

Craving for physical activity

Physical activity craving was assessed with the following item: "At this moment, to what extent do you feel the desire (urge) to engage in physical activity (e.g., running, exercising, moving)?". Participants respond on a scale from 1 "no desire at all" to 7 "very strong desire".

Supplementary Material 3. Results of the exploratory analyses for reaction times

Usual level of physical activity

Results of the linear mixed-effects models predicting the reaction times as a function of the type of action (approach vs. avoidance) and of the type of stimuli (Running vs. Standing vs. Sitting) and usual level of physical activity

Moderation by the usual level of physical activity (N = 40)	b (CI)	*p*
**Fixed Effects**		
Intercept	**665.7 (627.2;704.2)**	**<.001**
**Stimuli (ref. Running)**		
Standing	2.7 (-11.8;17.3)	.712
Sitting	7.9 (-6.0;18.93)	.267
**Action (ref. approach)**		
Avoidance	-6.9 (-23.6;21.9)	.415
**Stimuli (ref. Running) x Action (ref. approach)**		
Avoidance x Standing	-4.1 (-22.5;14.2)	.661
Avoidance x Sitting	-1.7 (-20.1;16.6)	.852
**Usual level of physical activity**		
Usual level of physical activity	13.3 (-21.2;47.8)	.454
Usual level of physical activity x Sitting	-6.0 (-19.2;7.2)	.373
Usual level of physical activity x Standing	-2.4 (-15.4;10.6)	.718
Usual level of physical activity x Avoidance	-7.6 (-24.2;8.9)	.369
Usual level of physical activity x Standing x Avoidance	-1.4 (-19.8;16.9)	.877
Usual level of physical activity x Sitting x Avoidance	9.1 (-9.2;27.5)	.330
**Covariates**		
Age	-12.7 (-48.27;21.8)	.476
Sex	-3.9 (-73.27;70.3)	.918
Body mass index	-0.7 (-31.21;35.0)	.968
**Random Effects**		
**Participants**		
Intercept	10877.52	
Stimuli Sitting	55.82	
Stimuli standing	10.32	
Action Avoid	1130.10	
Corr. (Intercept, stimuli Sitting)	0.09	
Corr. (Intercept, stimuli standing)	0.96	
Corr. (Intercept, action avoidance)	-0.29	
Corr. (Stimuli Sitting; stimuli standing)	-0.19	
Corr. (Stimuli Sitting; action avoidance)	0.93	
Corr. (Stimuli standing; action avoidance)	-0.55	
**Stimuli**		
Intercept	95.08	
Residual	28558.12	
R^2	Conditional .006<br>Marginal =.280	

Notes. 95CI = confidence intervals at 95%.

Craving for sedentary behaviors

Results of the linear mixed-effects models predicting the reaction times as a function of the type of action (approach vs. avoidance) and of the type of stimuli (Running vs. Standing vs. Sitting) and craving for sedentary behaviors

**Moderation by the craving for sedentary behaviors (N = 40)**	b (CI)	*p*
**Fixed Effects**		
Intercept	**664.6 (626.1;703.2)**	**<.001**
**Stimuli (ref. Running)**		
Standing	2.8 (-11.7;17.3)	.706
Sitting	8.1 (-5.7;21.9)	.251
**Action (ref. approach)**		
Avoidance	-7.0 (-23.5;9.5)	.407
**Stimuli (ref. Running) x Action (ref. approach)**		
Avoidance x Standing	-4.2 (-22.5;14.2)	.565
Avoidance x Sitting	-2.1 (-20.4;16.3)	.826
**Craving for sedentary behaviors**		
Craving for sedentary behaviors	-3.2 (-37.0;30.5)	.852
Craving for sedentary behaviors x Sitting	**-22.0 (-35.0;-9.0)**	**<.001**
Craving for sedentary behaviors x Standing	-5.1 (-18.1;8.0)	.449
Craving for sedentary behaviors x Avoidance	-14.45 (-30.9;2.0)	.089
Craving for sedentary behaviors x Standing x Avoidance	3.0 (-153;21.36)	.747
Craving for sedentary behaviors x Sitting x Avoidance	15.3 (-3.0;33.65)	.102
**Covariates**		
Age	-11.4 (-45.60;20.67)	.514
Sex	-0.14 (-55.55;85.92)	.997
Body mass index	3.0 (-27.83;39.00)	.864
**Random Effects**		
**Participants**		
Intercept	10979.23	
Stimuli Sitting	11.29	
Stimuli standing	9.07	
Action Avoid	1092.45	
Corr. (Intercept, Sitting)	-0.30	
Corr. (Intercept, Standing)	0.84	
Corr. (Intercept, Avoidance)	-0.33	
Corr. (Sitting; Standing)	-0.77	
Corr. (Sitting; Avoidance)	1.00	
Corr. (Standing; Avoidance)	-0.79	
**Stimuli**		
Intercept	95.40	
Residual	28524.59	
R^2	Conditional = .012<br>Marginal = .280	

Notes. 95CI = confidence intervals at 95%.

Results of the linear mixed-effects models.

A. Region of significance of the difference in reaction times to respond to Sitting relative to Running stimuli as function of the level of craving for sedentary behaviors. B. Prediction of the response times as a function of the type of stimuli and the level of craving for sedentary behaviors.

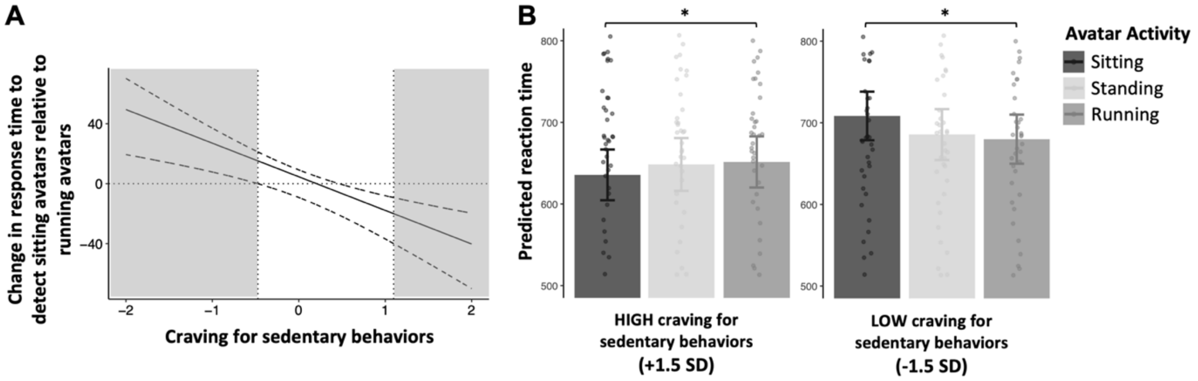

Notes. A. A positive effect (i.e., grey area on the left) indicates that participants were significantly slower to respond to Sitting relative to Running stimuli, while a negative effect (i.e., grey area on the right) indicates the contrary – participants were significantly quicker to respond to Sitting relative to Running stimuli. Craving for sedentary behaviors are standardized. B. Dots representing mean response times for each participant as a function of the type of stimuli (i.e., Running vs. Sitting vs. Standing stimuli). Errors bars represent the standard errors around the mean.

Craving for physical activity

Results of the linear mixed-effects models predicting the reaction times as a function of the type of action (approach vs. avoidance) and of the type of stimuli (Running vs. Standing vs. Sitting) and craving for physical activity

**Moderation by the craving for physical activity (N = 40)**	b (CI)	*p*
**Fixed Effects**		
Intercept	**665.8 (627.0;704.5)**	**<.001**
**Stimuli (ref. Running)**		
Standing	2.8 (-11.7;17.3)	.707
Sitting	8.0 (-6.0;21.9)	.264
**Action (ref. approach)**		
Avoidance	-6.9 (-23.6;9.8)	.418
**Stimuli (ref. Running) x Action (ref. approach)**		
Avoidance x Standing	-4.2 (-22.5;14.2)	.656
Avoidance x Sitting	-1.9 (-20.2;16.5)	.842
**Craving for physical activity**		
Craving for physical activity	-8.8 (-43.2;25.7)	.919
Craving for physical activity x Sitting	9.5 (-3.7;22.7)	.159
Craving for physical activity x Standing	8.2 (-4.9;21.2)	.218
Craving for physical activity x Avoidance	2.5 (-14.2;19.2)	.773
Craving for physical activity x Standing x Avoidance	-8.0 (-26.3;10.3)	.392
Craving for physical activity x Sitting x Avoidance	-1.2 (-19.6;17.2)	.899
**Covariates**		
Age	-13.3 (-48.3;20.67)	.460
Sex	-4.1 (-78.6;85.92)	.914
Body mass index	1.8 (-33.1;39.00)	.918
**Random Effects**		
**Participants**		
Intercept	10976.35	
Stimuli Sitting	56.98	
Stimuli standing	8.9	
Action Avoid	1158.77	
Corr. (Intercept, Sitting)	0.09	
Corr. (Intercept, Standing)	0.98	
Corr. (Intercept, Avoidance)	-0.30	
Corr. (Sitting; Standing)	-0.13	
Corr. (Sitting; Avoidance)	0.93	
Corr. (Standing; Avoidance)	-0.50	
**Stimuli**		
Intercept	94.44	
Residual	28549.61	
R^2	Conditional = .005<br>Marginal = .281	

Notes. 95CI = confidence intervals at 95%.

Supplementary Material 4. Results of the exploratory analyses for errors

Usual level of physical activity

Results of the logistic mixed-effects models predicting the risk of error in the approach-avoidance task as a function of action type (approach vs. avoidance) and stimuli type (Running vs. Standing vs. Sitting), and usual physical activity.

N = 40	OR (CI)	*p*
**Fixed Effects**		
Intercept	**0.06 (0.04;0.08)**	**<.001**
**Stimuli (ref. Running)**		
Standing	0.80 (0.59;1.09)	.151
Sitting	0.85 (0.63;1.15)	.294
**Action (ref. approach)**		
Avoidance	0.73 (0.52;1.03)	.070
**Stimuli (ref. Running) x Action (ref. approach)**		
Avoidance x Standing	**1.57 (1.01;2.44)**	**.045**
Avoidance x Sitting	**1.64 (1.06;2.54)**	**.025**
**Usual level of physical activity**		
Usual level of physical activity	1.03 (0.72;1.45)	.887
Usual level of physical activity x Sitting	1.02 (0.74;1.40)	.906
Usual level of physical activity x Standing	0.96 (0.70;1.33)	.814
Usual level of physical activity x Avoidance	0.91 (0.64;1.28)	.575
Usual level of physical activity x Standing x Avoidance	1.05 (0.66;1.66)	.850
Usual level of physical activity x Sitting x Avoidance	1.10 (0.70;1.74)	.669
**Covariates**		
Age	1.04 (0.77;1.40)	.808
Sex	0.95 (0.49;1.83)	. 881
Body mass index	0.91 (0.66;1.24)	.542
**Random Effects**		
**Participants**		
Intercept	0.68	
Stimuli Sitting	0.01	
Stimuli standing	0.02	
Action Avoid	0.10	
Corr. (Intercept, Sitting)	0.79	
Corr. (Intercept, Standing)	0.99	
Corr. (Intercept, Avoidance)	-0.39	
Corr. (Sitting; Standing)	0.71	
Corr. (Sitting; Avoidance)	-0.87	
Corr. (Standing; Avoidance)	-0.27	
**Stimuli**		
Intercept	Null	
R^2	Conditional = .006<br>Marginal = .192	

Notes. OR= odds ratio; 95CI = confidence intervals at 95%. Note that the models estimated a null variance for the random intercept of the stimuli. The models with or without this parameter lead to consistent results. *Two participants were not included in the analyses because they were an issue regarding the recording of their behavioral data.

Craving for sedentary behaviors

Results of the logistic mixed-effects models predicting the risk of error in the approach-avoidance task as a function of action type (approach vs. avoidance) and stimuli type (Running vs. Standing vs. Sitting), and craving for sedentary behaviors.

**N = 40**	OR (CI)	*p*
**Fixed Effects**		
Intercept	**0.06 (0.04;0.08)**	**<.001**
**Stimuli (ref. Running)**		
Standing	0.82 (0.60;1.11)	.193
Sitting	0.82 (0.61;1.11)	.224
**Action (ref. approach)**		
Avoidance	0.74 (0.53;1.03)	.069
**Stimuli (ref. Running) x Action (ref. approach)**		
Avoidance x Standing	**1.49 (0.96;2.31)**	**.018**
Avoidance x Sitting	**1.70 (1.10;2.63)**	**.079**
**Craving for sedentary behaviors**		
Craving for sedentary behaviors	0.80 (0.57;1.13)	.180
Craving for sedentary behaviors x Sitting	0.84 (0.61;1.15)	.245
Craving for sedentary behaviors x Standing	1.03 (0.75;1.42)	.854
Craving for sedentary behaviors x Avoidance	1.16 (0.82;1.65)	.345
Craving for sedentary behaviors x Standing x Avoidance	0.73 (0.46;1.15)	.125
Craving for sedentary behaviors x Sitting x Avoidance	1.26 (0.80;1.98)	.276
**Covariates**		
Age	1.04 (0.77;1.40)	.800
Sex	0.94 (0.49;1.82)	.864
Body mass index	0.92 (0.67;1.26)	.593
**Random Effects**		
**Participants**		
Intercept	0.64	
Stimuli Sitting	0.01	
Stimuli standing	0.02	
Action Avoid	0.08	
Corr. (Intercept, Sitting)	0.59	
Corr. (Intercept, Standing)	0.98	
Corr. (Intercept, Avoidance)	-0.31	
Corr. (Sitting; Standing)	0.42	
Corr. (Sitting; Avoidance)	-0.95	
Corr. (Standing; Avoidance)	-0.11	
**Stimuli**		
Intercept	Null	
R^2	Conditional = .021<br>Marginal = .196	

Notes. OR= odds ratio; 95CI = confidence intervals at 95%. Note that the models estimated a null variance for the random intercept of the stimuli. The models with or without this parameter lead to consistent results. *Two participants were not included in the analyses because they were an issue regarding the recording of their behavioral data.

Craving for physical activity

Results of the logistic mixed-effects models predicting the risk of error in the approach-avoidance task as a function of action type (approach vs. avoidance) and stimuli type (Running vs. Standing vs. Sitting), and craving for physical activity.

**N = 40**	OR (CI)	*p*
**Fixed Effects**		
Intercept	**0.6 (0.04;0.08)**	**<.001**
**Stimuli (ref. Running)**		
Standing	0.78 (0.57;1.06)	.117
Sitting	0.85 (0.62;1.15)	.289
**Action (ref. approach)**		
Avoidance	0.71 (0.51;1.00)	.052
**Stimuli (ref. Running) x Action (ref. approach)**		
Avoidance x Standing	**1.67 (1.07;2.61)**	**.024**
Avoidance x Sitting	**1.65 (1.06;2.59)**	**.027**
**Craving for physical activity**		
Craving for physical activity	0.96 (0.69;1.34)	.799
Craving for physical activity x Sitting	0.83 (0.62;1.12)	.218
Craving for physical activity x Standing	0.86 (0.64;1.16)	.331
Craving for physical activity x Avoidance	0.77 (0.55;1.07)	.119
Craving for physical activity x Standing x Avoidance	1.43 (0.94;2.19)	.097
Craving for physical activity x Sitting x Avoidance	1.11 (0.73;1.70)	.620
**Covariates**		
Age	1.04 (0.74;1.33)	.948
Sex	0.94 (0.53;1.89)	.997
Body mass index	0.92 (0.69;1.25)	.610
**Random Effects**		
**Participants**		
Intercept	0.67	
Stimuli Sitting	0.01	
Stimuli standing	0.02	
Action Avoid	0.10	
Corr. (Intercept, Sitting)	0.40	
Corr. (Intercept, Standing)	1.00	
Corr. (Intercept, Avoidance)	-0.44	
Corr. (Sitting; Standing)	0.40	
Corr. (Sitting; Avoidance)	-1.00	
Corr. (Standing; Avoidance)	-0.43	
**Stimuli**		
Intercept	Null	
R^2	Conditional = .020<br>Marginal = .195	

Notes. OR= odds ratio; 95CI = confidence intervals at 95%. Note that the models estimated a null variance for the random intercept of the stimuli. The models with or without this parameter lead to consistent results. *Two participants were not included in the analyses because they were an issue regarding the recording of their behavioral data.

Supplementary Material 5. Neural activity associated with the avoidance of sitting stimuli: Avoid Sitting > Avoid Standing

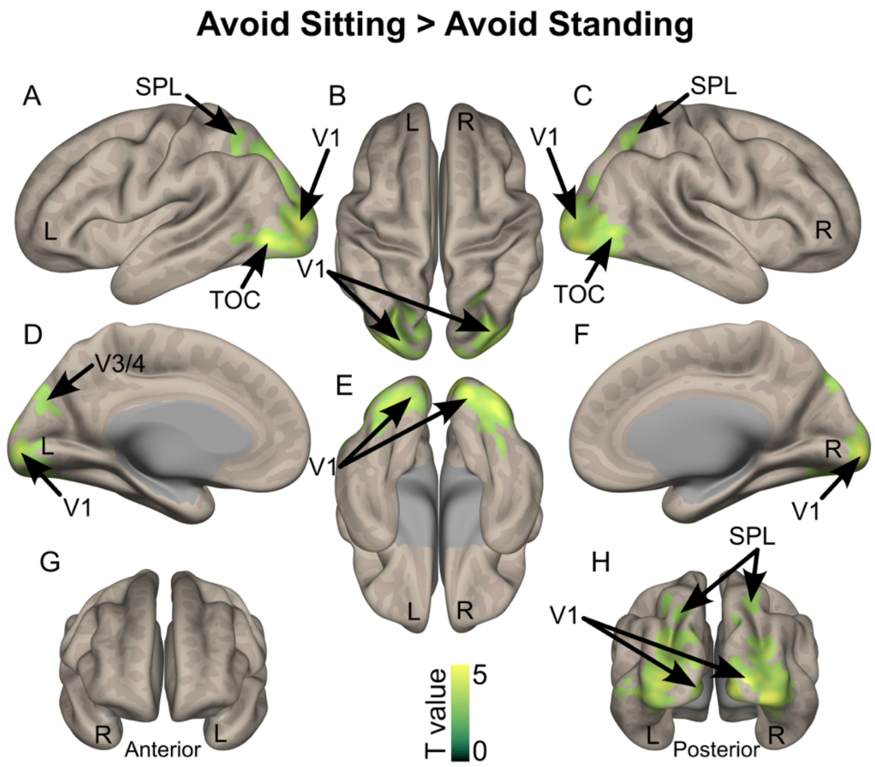

Brain activations when avoiding Sitting vs. Standing stimuli, corrected for multiple comparisons (whole-brain voxel-wise *p* < .05 FDR, k > 10 voxels). The color bar represents the statistical T value. SPL: superior parietal lobule; TOC: temporo-occipital cortex; V1: primary visual cortex; V3/4: associative visual cortex. L: left hemisphere; R: right hemisphere.

Supplementary Material 6. Detailed coordinates of the clusters presented in this section.

MNI coordinates for the neuroimaging results of the Approach Sitting vs. Avoid Sitting contrast

Region	Hemisphere	Cluster size	T value	MNI X	MNI Y	MNI Z
Fusiform cortex	R	1594	5,65	28	-56	-8
Mid Occipital cortex (V1)	L		5,34	-14	-94	0
Fusiform cortex	R		5,24	28	-76	-12
Mid Occipital cortex (V1)	R		4,73	18	-84	-10
Inf Occipital cortex (V2/3)	R		4,69	36	-80	-12
Parahippocampal gyrus	L	159	4,58	-26	-44	-8
Fusiform cortex	L	31	4,34	-36	-40	-22
Mid Occipital cortex	L	92	4,20	-30	-78	24
Mid Occipital cortex	L		3,71	-26	-84	14
Mid Occipital cortex	L		3,48	-38	-80	18
Mid Occipital cortex	R	60	4,15	32	-76	20
Thalamus	R	12	3,76	24	-26	-6
Parahippocampal gyrus	L	11	3,64	-20	-32	-8

V1: primary visual cortex; V2/3: secondary visual cortex. MNI: Montreal Neurological Institute.

MNI coordinates for the neuroimaging results of the Avoid Sitting vs. Approach Sitting contrast

Region	Hemisphere	Cluster size	T value	MNI X	MNI Y	MNI Z
SMA	R	1356	4,85	2	-18	66
Mid Cingulate cortex	R		4,73	8	-32	54
SMA	R		4,72	10	-24	58
Mid Cingulate cortex	R		4,71	6	-16	46
Primary motor cortex	R		4,61	28	-30	60
Insula	R	213	4,49	46	8	4
IFG tri	R		3,94	46	24	2
IFG oper	R		3,92	46	16	0
IFG orb	R		3,66	54	22	-4
DLPFC	R	227	4,47	28	44	18
DLPFC	R		4,25	34	50	18
DLPFC	R		3,71	26	38	26
Primary motor cortex	L	275	4,35	-20	-26	66
S1	L		4,32	-22	-34	56
S1	L		3,57	-32	-42	58
STG post	R	94	4,19	48	-24	14
STS mid	R	209	4,16	50	-32	0
STG post	R		4,13	54	-24	-2
Temporal pole	R	41	4,11	46	22	-18
Primary motor cortex	L	182	4,07	-50	-8	46
Pre primary motor cortex	R	122	4,06	44	-6	40
IFG oper	L	59	3,95	-50	6	2
Insula	L	46	3,95	-42	4	10
Frontal gyrus mid	L	61	3,91	-28	52	6
Cingulate cortex mid	R	24	3,91	10	18	42
Cingulate cortex mid	R	42	3,90	10	-4	38
Heschl gyrus	L	70	3,85	-34	-26	6
Planum temporale	L		3,84	-42	-22	8
Cerebellum lobule 6	L	14	3,70	-26	-44	-34
Thalamus	L	26	3,68	-22	-12	14
Putamen	L		3,31	-28	-8	10
Insula	L	19	3,65	44	-6	10

SMA: supplementary motor area; IFG: inferior frontal gyrus (tri: *pars triangularis*, oper: *pars opercularis*, orb: *pars orbitalis*); DLPFC: dorsolateral prefrontal cortex; S1: primary somatosensory cortex; STG: superior temporal gyrus; STS: superior temporal sulcus; post: posterior.

MNI coordinates for the neuroimaging results of the Avoid Sitting vs Avoid Running contrast

Region	Hemisphere	Cluster size	T value	MNI X	MNI Y	MNI Z
STG mid	R	614	5,01	50	-6	-6
STG mid	R		4,84	52	-18	-6
STS post	R		4,50	50	-34	-2
STG post	R		4,47	66	-44	16
Thalamus	R	145	4,80	4	-12	4
Thalamus	R		4,01	12	-26	-2
Substania nigra	R		3,60	14	-18	-8
SMA	L	78	4,72	-14	-24	66
Cerebellum lobule 4-5	R	80	4,37	10	-56	-20
Cerebellum lobule 4-6	R		3,82	10	-44	-12
Globus pallidus	L	48	4,34	-20	0	-2
Putamen	L		3,57	-32	0	-4
Temporal pole	L	54	4,26	-50	12	-20
Globus pallidus	R	118	4,20	30	-10	-4
Globus pallidus	R		4,05	20	-6	2
Putamen	R		3,81	28	-8	6
Cerebellum Crus 1	L	58	4,10	-18	-68	-28
Cerebellum lobule 6	R	12	3,96	16	-70	-26
STG post	L	21	3,93	-54	-46	20
DLPFC	R	51	3,91	28	48	20
Insula	R	30	3,87	-34	8	-8
Cingulate cortex mid	L	13	3,86	8	-18	44
MTG ant	L	14	3,82	-56	-6	-24
Angular gyrus	L	16	3,61	-50	-66	24

STG: superior temporal gyrus; STS: superior temporal sulcus (post: posterior); SMA: supplementary motor area; DLPFC: dorsolateral prefrontal cortex; MTG: middle temporal gyrus (ant: anterior).

MNI coordinates for the neuroimaging results of the Avoid Sitting vs. Avoid Standing contrast.

Region	Hemisphere	Cluster size	T value	MNI X	MNI Y	MNI Z
Calcarine (V1)	R	2991	5,70	20	-94	2
Occipital cortex mid	R		5,34	38	-82	-6
Lingual gyrus	R		5,13	18	-90	-8
Occipital cortex inf	R		5,08	30	-86	-4
Occipital cortex sup	L	3013	5,20	-12	-96	6
Occipital cortex mid	L		5,12	-30	-76	24
Occipital cortex mid	L		5,11	-24	-88	14
Parietal lobule sup	L	77	3,76	-20	-68	48
Parietal lobule sup	L		3,22	-22	-62	42
Cuneus	R	37	3,69	16	-80	42
Parietal lobule sup	R	166	3,67	26	-72	52
Parietal lobule sup	R		3,67	26	-60	56
Parietal lobule inf	L	36	3,65	-28	-54	42

V1: primary visual cortex; sup: superior; inf: inferior.
